# Identification and mechanism of G protein-biased ligands for chemokine receptor CCR1

**DOI:** 10.1038/s41589-021-00918-z

**Published:** 2021-12-23

**Authors:** Zhehua Shao, Qingya Shen, Bingpeng Yao, Chunyou Mao, Li-Nan Chen, Huibing Zhang, Dan-Dan Shen, Chao Zhang, Weijie Li, Xufei Du, Fei Li, Honglei Ma, Zhi-Hua Chen, H. Eric Xu, Songmin Ying, Yan Zhang, Huahao Shen

**Affiliations:** 1 Key Laboratory of Respiratory Disease of Zhejiang Province, Department of Respiratory and Critical Care Medicine, Second Affiliated Hospital of Zhejiang University School of Medicine, Hangzhou, China; 2 Department of Biophysics and Department of Pathology of Sir Run Run Shaw Hospital, Zhejiang University School of Medicine, Hangzhou, China; 3 Liangzhu Laboratory, Zhejiang University Medical Center, Hangzhou, China; 4 Department of Pharmacology and Department of Respiratory and Critical Care Medicine of the Second Affiliated Hospital, Zhejiang University School of Medicine, Key Laboratory of Respiratory Disease of Zhejiang Province, Hangzhou, China; 5 Department of Anatomy, Zhejiang University School of Medicine, Hangzhou, China; 6 The CAS Key Laboratory of Receptor Research, Shanghai Institute of Materia Medica, Chinese Academy of Sciences, Shanghai, China; 7 University of Chinese Academy of Sciences, Beijing, China; 8 School of Life Science and Technology, ShanghaiTech University, Shanghai, China; 9 International Institutes of Medicine, the Fourth Affiliated Hospital of Zhejiang University School of Medicine, Yiwu, China; 10 Zhejiang Provincial Key Laboratory of Immunity and Inflammatory Diseases, Hangzhou, China; 11 MOE Frontier Science Center for Brain Research and Brain-Machine Integration, Zhejiang University School of Medicine, Hangzhou, China; 12 State Key Laboratory of Respiratory Disease, Guangzhou, China

**Keywords:** X-ray crystallography, Peptides, G protein-coupled receptors, Cell signalling

## Abstract

Biased signaling of G protein-coupled receptors describes an ability of different ligands that preferentially activate an alternative downstream signaling pathway. In this work, we identified and characterized different N-terminal truncations of endogenous chemokine CCL15 as balanced or biased agonists targeting CCR1, and presented three cryogenic-electron microscopy structures of the CCR1–G_i_ complex in the ligand-free form or bound to different CCL15 truncations with a resolution of 2.6–2.9 Å, illustrating the structural basis of natural biased signaling that initiates an inflammation response. Complemented with pharmacological and computational studies, these structures revealed it was the conformational change of Tyr291 (Y291^7.43^) in CCR1 that triggered its polar network rearrangement in the orthosteric binding pocket and allosterically regulated the activation of β-arrestin signaling. Our structure of CCL15-bound CCR1 also exhibited a critical site for ligand binding distinct from many other chemokine–receptor complexes, providing new insights into the mode of chemokine recognition.

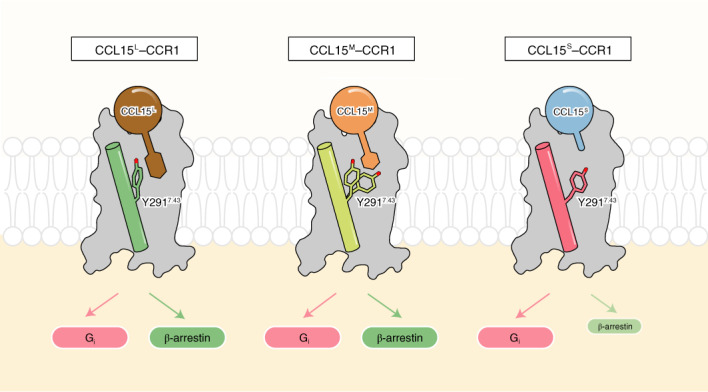

## Abstract (web summary)

Cryo-EM structures of the CCR1–G_i_ complex with N-terminal truncations of the endogenous chemokine CCL15 revealed that the conformational change of Tyr291 in CCR1 regulates β-arrestin signaling.

## Main

G protein-coupled receptors (GPCRs) constitute the largest family of transmembrane proteins that transmit extracellular stimulus across the plasma membrane and initiate cellular signaling pathways via coupling to heterotrimeric G proteins or β-arrestin^1,2^. In most cases, the activation of G proteins and β-arrestin corresponds to their own respective downstream effectors, thus performing distinct biological functions^3^. Specific ligands at some GPCRs, such as the type 1 angiotensin II receptor (AT_1_R), the neurotensin receptor 1 (NTSR1), the μ-opioid receptor (μOR) and the kappa opioid receptor (κOR), have been demonstrated to preferentially stimulate the G protein or β‐arrestin signaling pathways^4–7^. This phenomenon is termed ‘biased signaling’, provides new insights into GPCR biology and has a broad prospect of clinical applications, since selective activation or inhibition of specific signaling cascades has been proved to increase the therapeutic use of drugs targeting GPCRs with minimal side effects. Nevertheless, understanding of molecular mechanism underlining biased signaling has so far remained a challenging task^3^.

**Extended Data Fig. 1:**
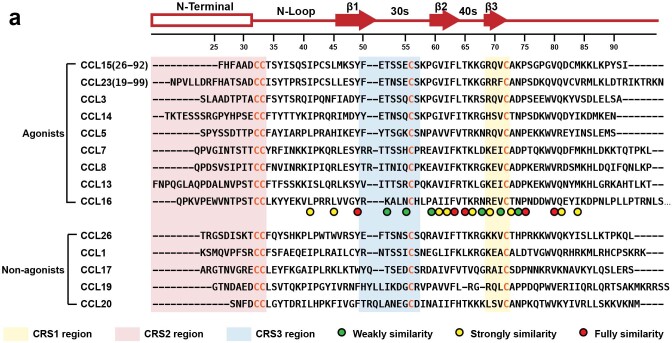
Sequence alignment of CC chemokines. **a**, Sequence alignment of nine human endogenous CCR1 agonists and other CCR1 non-agonists. The sequences of CRS1, CRS2 and CRS3 regions were highlighted in yellow, red and blue, respectively. And the positions which share fully conserved residues were marked with red circles. Residues with strongly similar properties (scoring > 0.5 in the Gonnet PAM 250 matrix) were marked with yellow circles. Residues with weakly similar properties (scoring < 0.5 in the Gonnet PAM 250 matrix) were marked with green circles.

Chemokine–receptor interactions play an essential role in guiding leukocyte trafficking in immune surveillance and inflammation response^8,9^. According to the number and position of conserved cysteine residues in their N-terminal regions, chemokines are classified as cysteine, cysteine cysteine, cysteine X cysteine (CXC) and cysteine X3 cysteines (CX3C) subsets^10–12^. So far, at least 50 endogenous chemokines and 20 chemokine receptors have been identified in humans. The interactions between chemokines and their receptors exhibit considerable promiscuity, wherein most receptors can be recognized by multiple chemokines and most chemokines can activate multiple receptors. These chemokine–receptor interactions were previously regarded as redundant, but it is now appreciated that many chemokine interactions display biased agonism, enabling the fine-tuning of a chemokine-induced physiological response^3,13^. Among the chemokine receptor family, the chemokine receptor CCR1 unusually exhibits the most ligand promiscuity, which can recognize at least nine human cysteine cysteine chemokines, including CCL3, CCL5-9 (CCL6 and CCL9 are murine), CCL13–16 and CCL23 (refs. ^9,11,14^). CCR1 is widely expressed in various immune cells, and the knockdown of CCR1 has proved effective in suppressing the maturation and migration of immune cells, thus being regarded as an attractive drug target for the treatment of many autoimmune and allergic diseases, such as asthma^15–18^. Structure-based sequence alignment of CCR1 agonists shows high divergences at the N terminus, with the core region exhibiting conserved similarity, indicating that the N terminus of chemokines contributes to different signal transduction properties of CCR1 (Extended Data Fig. 1a).

CCL15 is an endogenous ligand of CCR1 at which the N-terminal signal peptide is cleaved up on its secretion to extracellular media. Identified by mass spectrometry of clinical samples, the N terminus of secreted CCL15 underwent further cleavage and exhibited different N-terminal truncations due to proteolytic processing mediated by activated mast cells and neutrophils^19,20^. For instance, CCL15 (22–92) was generated after digestion by either chymase or cathepsin G, while CCL15 (29–92) was produced by elastase treatment of CCL15 (ref. ^19^). More and more studies have suggested that the N terminus of chemokines worked as a determinant for CCR1 activation, as well as giving rise to biased agonism^21,22^. In this study, we found that compared to the longer form of CCL15 N-terminal truncation (CCL15 (26–92), termed CCL15^L^), the shorter forms of CCL15 N-terminal truncations (CCL15 (27–92), termed CCL15^M^, and CCL15 from (28–92) to (31–92), termed CCL15^S^) displayed poor efficacy in β-arrestin recruitment, thus performing stronger bias toward G protein pathways. To develop an understanding of the structural features that contributes to the biased signaling of CCR1, we determined three cryogenic-electron microscopy (cryo-EM) structures of human CCR1–G_i_ complexes either in the absence of ligand (apo) or in bound to CCL15^L^ or CCL15^M^. Together with mutagenesis and functional studies, our results provide a new molecular mechanism to explain biased signaling of CCR1 and reveal the diverse binding modes of chemokine receptors that contribute to ligand-selective recognition.

## Results

### Biased signaling of CCR1 induced by CCL15

**Fig. 1:**
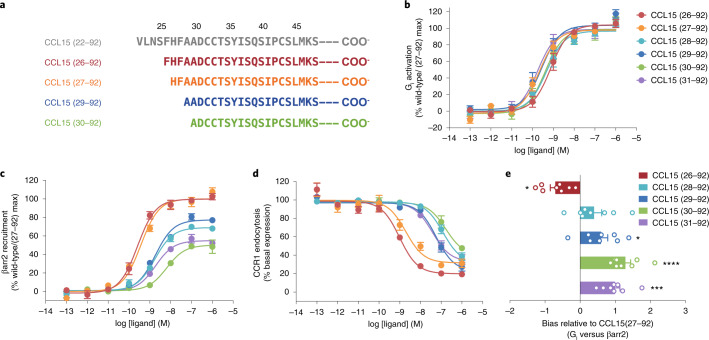
Natural biased agonism by CCL15 variants. **a**, The N-terminal sequences of CCL15 truncations identified by mass spectrometry. **b**,**c**, The effects of different CCL15 N-terminal truncations on CCR1. Dose–response curves for CCL15-induced G_i1_ signaling (**b**) and β-arrestin2 recruitment (**c**) were measured by NanoBiT assay. *N* = eight independent experiments, performed with single replicates. **d**, Dose–response curves of CCR1 endocytosis on THP-1 cells measured by the relative fluorescence intensity. NanoBiT data were normalized to the maximal response of CCL15 (27–92) on CCR1 (wild-type), and the percentage response in the endocytosis of CCR1 was normalized to cells without ligand treatment. *N* = six independent experiments, performed with single replicates. **e**, Bias factors of CCL15 truncations relative to CCL15 (27–92). Bias factors derived from curve fit parameters from **b**,**c**. The asterisk symbols indicated statistically significant difference (*P* = 0.015, *P* = 0.2682, *P* = 0.0426, *P* < 0.0001 and *P* = 0.0003 from top to bottom, *P < 0.05, ***P < 0.001, ****P < 0.0001) for CCL15 truncations versus CCL15 (27–92), determined by one-way ANOVA. *N* = 8 independent experiments, performed with single replicates. In **d**,**e**, all data are shown as mean ± s.e.m. Source data

**Extended Data Fig. 2:**
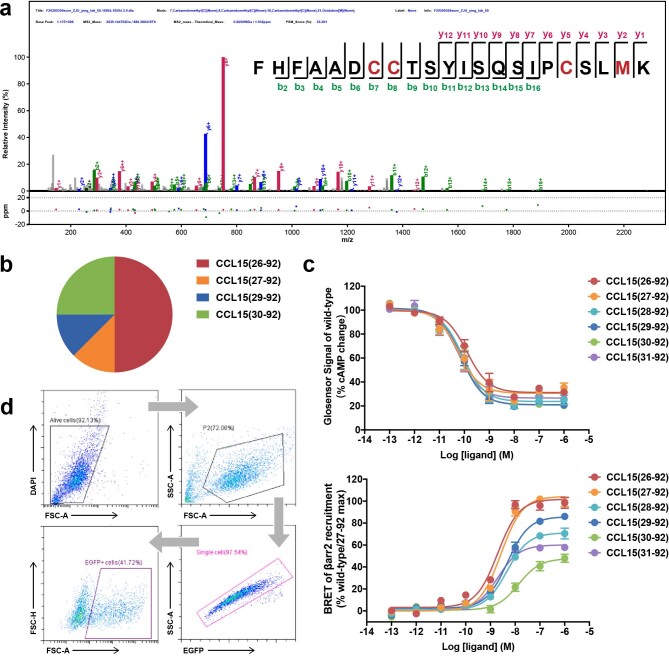
Comparisons among different N-terminal truncations of CCL15 in biased signaling. **a**, The major form of N-terminal protein sequencing of the CCL15 involved in CCR1-G_i_ complex verified by the mass spectrometry. **b**, The proportions of CCL15 N-terminal truncations identified in CCL15-CCR1-G_i_ complex. **c**, The effects of different CCL15 N-terminal truncations on CCR1. Dose-response curves for CCL15-induced G_i1_ signaling measured by Glosensor assay (upper, n= six independent experiments, performed with single replicates) and curves for β-arrestin signaling measured by BRET assay (lower, n= eight independent experiments, performed with single replicates) were shown; Data were shown as mean±SEM. **d**, Representative flow cytometry plots of flow analysis, illustrating the gating strategy for the endocytosis assay of HEK293T cells overexpresed CCR1. The endocytosis assay of THP-1 cells only included the first two shown steps. Source data

To study the mechanism for CCL15-mediated activation of CCR1, we purified CCL15 (22–92) from insect cells, which was reported as the longest N-terminal digestion product^19^. Identified by mass spectrometry, we found that the first determined residue of the CCL15 N terminus in the G_i_-coupled complex was F26, and the main forms of CCL15 truncations involved in complex formation were CCL15 (26–92), CCL15 (27–92), CCL15 (29–92) and CCL15 (30–92) (Fig. 1a and Extended Data Fig. 2a,b). This phenomenon is consistent with the fact that physiological fluids, proinflammatory proteases or human cell supernatants can truncate the N termini of the cognate chemokines of CCR1, such as CCL6, CCL9, CCL15 and CCL23, thereby enabling the cleaved ligands to activate the receptor^19,20^.

Therefore, we separately purified N-terminal truncations of CCL15 from (26–92) to (31–92) and measured their effects on both G proteins and β-arrestin pathways. All N-terminal truncations of CCL15 exhibited almost the same or with only slightly reduced potency on G protein activation, but removal of the first two N-terminal amino acids, CCL15 truncations from (28–92) to (31–92), generated a strong decline in β-arrestin recruitment (Fig. 1b,c and Extended Data Fig. 2c). These results were in good agreement with the endocytosis of CCR1 in human monocytic THP-1 cells treated with corresponding N-terminal truncations, which was proposed to be mediated by β-arrestin pathway (Fig. 1d and Extended Data Fig. 2d)^1^. Thus, compared with CCL15 (26–92) and CCL15 (27–92), all the shorter variants, from CCL15 (28–92) to CCL15 (31–92), exhibited strong bias toward G_i_-protein pathway (Fig. 1e and Supplementary Table 1). These results were consistent with previous reports on CCR1 that CCL15 (29–92) was biased toward both Gα_i_ activation and cAMP inhibition relative to CCL15 (27–92)^22^.

Based on the above results, we anticipated that the N terminus of CCL15 was critical for β-arrestin recruitment when these truncations displayed low nanomolar potency albeit with reduced efficacy. Therefore, we grouped these N-terminal truncations of CCL15 into three categories: (1) CCL15 (26–92), which was a long form CCL15 (CCL15^L^) and showed a bias toward β-arrestin pathway; (2) CCL15 (27–92), which was a medium form CCL15 (CCL15^M^) and displayed balanced activation between G protein and β-arrestin pathways and (3) CCL15 truncations from (28–92) to (31–92), which were short forms of CCL15 (CCL15^S^) and were the strong G protein-biased agonists of CCR1. These findings revealed that the N-terminal region of CCL15 was not essential for CCR1-mediated G protein signaling, but critical for β-arrestin recruitment, suggesting the natural biased agonism of chemokine receptors under physiological conditions.

### Cryo-EM structures of three CCR1–G_i_ complexes

**Fig. 2:**
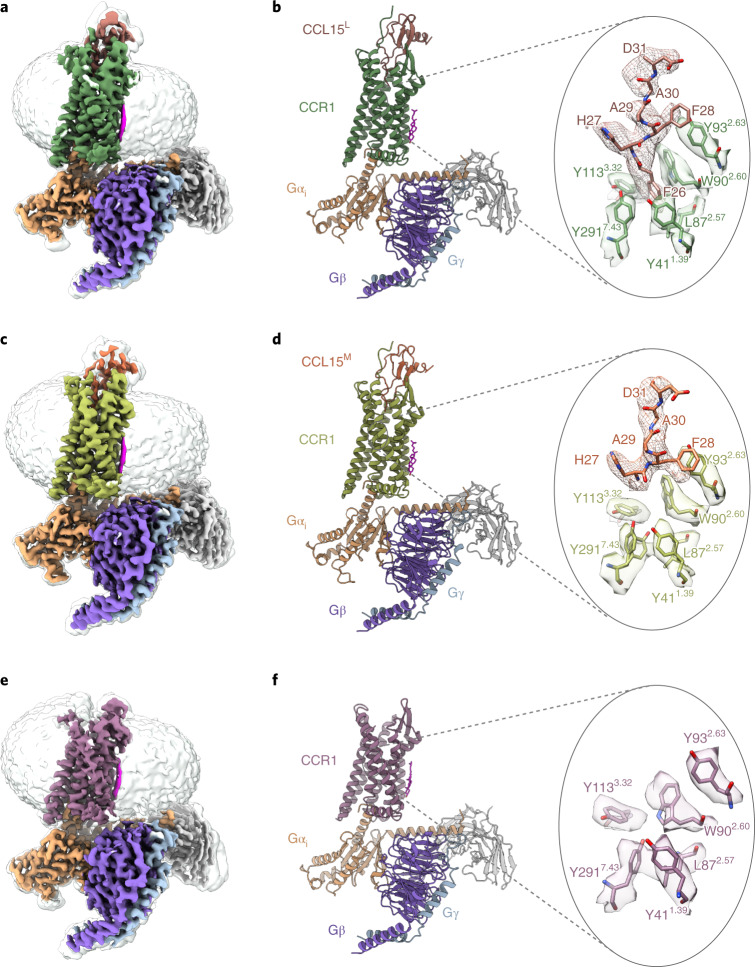
Cryo-EM structures of CCR1–G_i_ complexes in the CCL15^L^-, CCL15^M^- bound and apo states. **a**,**c**,**e**, Cryo-EM density maps of CCR1-G_i_ complexes bound to CCL15^L^ (**a**), CCL15^M^(**c**) and in the apo state (**e**). **b**,**d**,**f**, Models of CCR1–G_i_ complexes in three states (left). Sandy brown, Gα_i_; purple, Gβ; sky blue, Gγ; gray, scFv16 and green, CCL15^L^-bound CCR1 (**b**); brown, CCL15^L^ (**b**); dark khaki, CCL15^M^-bound CCR1 (**d**); orange, CCL15^M^ (**d**) and plum, apo-state CCR1. Cryo-EM maps and atomic resolution models showing the binding pockets in the 7TM domains of two CCR1–G_i_ complexes (right). Atomic models and EM density shown as sticks and surfaces, respectively. EM density of CCL15^L^ and CCL15^M^ shown as a mesh.

**Extended Data Fig. 3:**
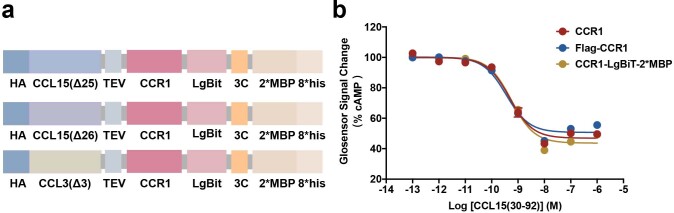
Single particle cryo-EM analysis of CCR1-G_i_ complexes in the apo and CCL15^M^-bound states. **a**, Schematic diagrams of human CCR1-related constructs used in this study. **b**, Glosensor cAMP responses of CCR1 on wild-type and modified receptors respectively. N= four independent experiments, performed with single replicates. Data were shown as mean±SEM. Source data

**Extended Data Fig. 4:**
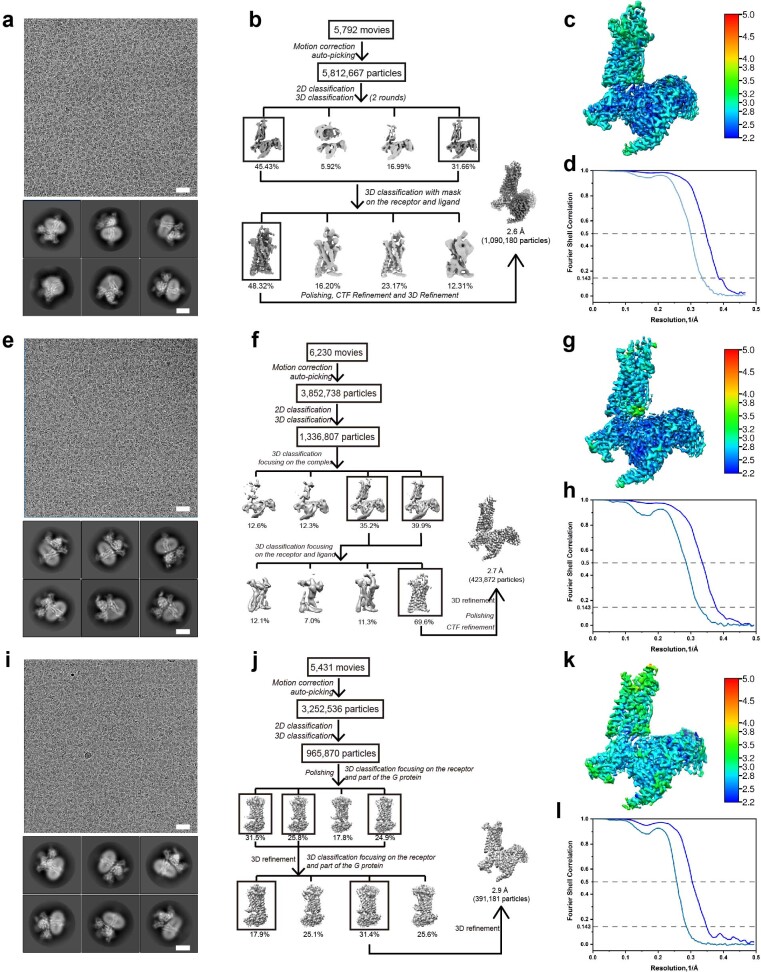
Cryo-EM maps and refined structures. **a, e, i**, Representative cryo-EM micrographs (scale bar: 30 nm) of the CCL15^L^-CCR1-G_i_ (**a**), CCL15^M^-CCR1-G_i_ (**e**), and apo CCR1-G_i_ (**i**) complexes (upper) and representative 2D class (bottom) averages showing distinct secondary structure features from different views (scale bar: 5 nm). **b, f, j**, Flowchart of cryo-EM data analysis for CCL15^L^-CCR1-G_i_ (**b**), CCL15^M^-CCR1-G_i_ (**f**), and apo CCR1-G_i_ (**j**) complexes. **c, g, k**, Cryo-EM maps of the CCR1-G_i_ complexes in CCL15^L^- (**c**), CCL15^M^-bound (**g**), and apo (**k**) states, colored by local resolution (Å) calculated using Bsoft package. **d, h, l**, ‘Gold-standard’ FSC curves corresponding to CCL15^L^-CCR1-G_i_ (**d**), CCL15^M^-CCR1-G_i_ (**h**), and apo CCR1-G_i_ (**l**) complexes.

**Extended Data Fig. 5:**
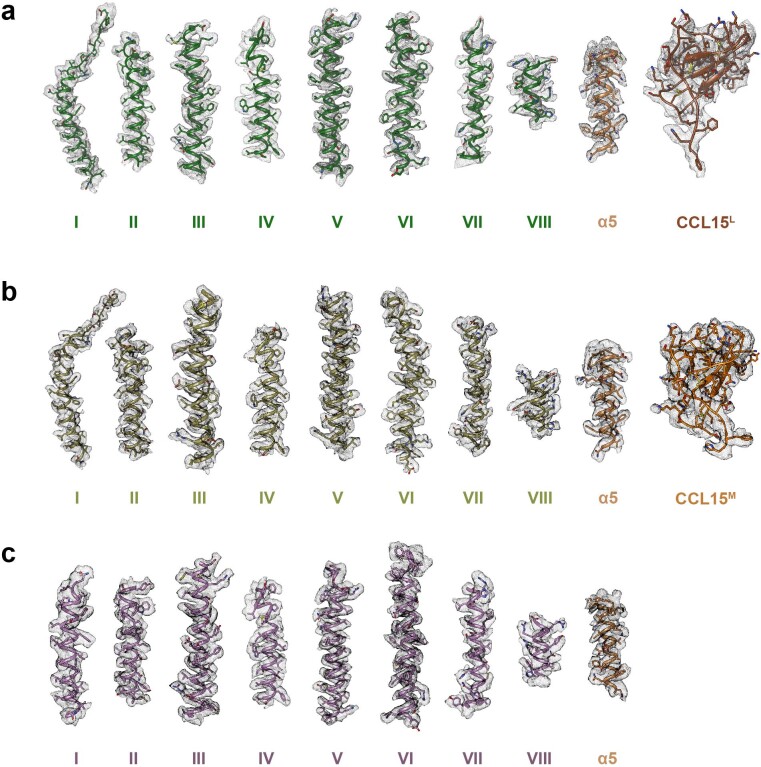
Structure comparison of CCR1 in CCL15^L^-, CCL15^M^-bound and apo states. **a**, Cryo-EM density maps and the models of CCL15^L^-CCR1-G_i_ complex were shown for all transmembrane helices, helix 8 of CCR1, helix 5 of Gα_i_ and CCL15^L^. **b**, Cryo-EM density maps and the models of CCL15^M^-CCR1-G_i_ complex were shown for all transmembrane helices, helix 8 of CCR1, helix 5 of Gα_i_ and CCL15^M^. **c**, Cryo-EM density maps and the models of apo CCR1-G_i_ complex were shown for all transmembrane helices, helix 8 of CCR1 and helix 5 of Gα_i_.

To investigate the molecular basis of signaling bias of CCR1, we aimed to obtain the structures of active CCR1 bound to CCL15 variants of different N-terminal truncations that displayed different bias profiles. The wild-type full-length human CCR1 and different ligands were coexpressed in insect cells for complex formation. A NanoBiT tethering strategy was used for complex stabilization (Extended Data Fig. 3)^23,24^. By using cryo-EM analyses, three structures of CCR1-G_i_ complexes in CCL15^L^-, CCL15^M^-bound and apo states were determined at overall resolutions of 2.6, 2.7 and 2.9 Å, respectively. Most side chains in the ligand and the seven transmembrane (7TM) domain of CCR1 in all three complexes could be clearly defined and enabled us to build and refine the near-atomic resolution structures of these complexes (Fig. 2 and Extended Data Figs. 4 and 5).

**Extended Data Fig. 6:**
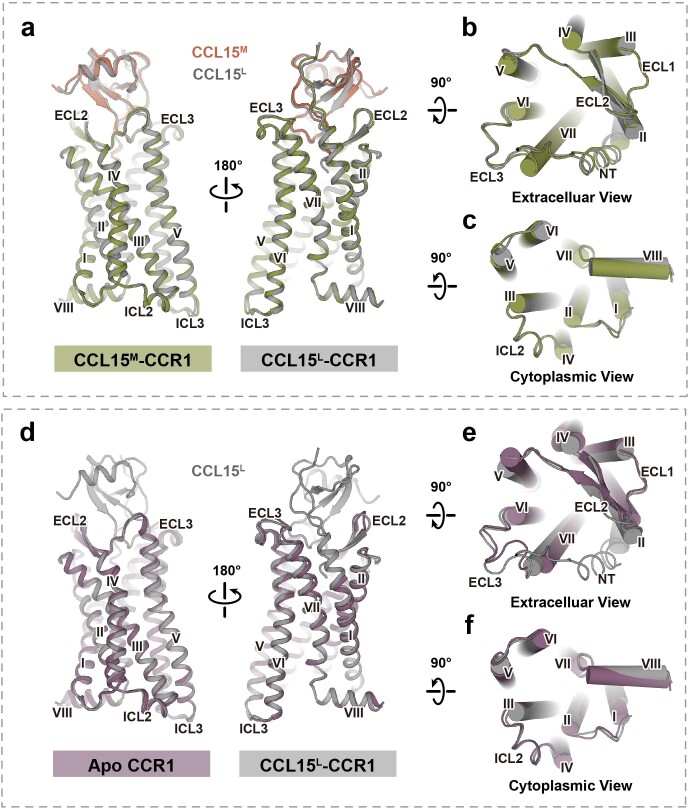
Structural comparison among chemokine-receptor complexes. **a-c**, Overlay of CCL15^M^ (sandy brown)-CCR1 (dark khaki) with CCL15^L^-CCR1 (gray) complex. Side (**a**), extracellular (**b**) and cytoplasmic (**c**) views were shown. **d-f**, Overlay of apo CCR1 (plum) with CCL15^L^-CCR1 (grey) complex. Side (**d**), extracellular (**e**) and cytoplasmic (**f**) views were shown.

Globally, all these CCR1 structures were similar to each other in their backbone conformations (Extended Data Fig. 6), suggesting a common conformation for G protein activation of CCR1 among these structures. In addition, the structure of the apo CCR1–G_i_ complex resembles the CCL15^L^-, CCL15^M^-bound CCR1–G_i_ complexes, except that for the bottom of the ligand-binding pockets in apo CCR1 was empty. The formation of the CCR1–G_i_ complex in the absence of the ligand was consistent with CCR1, which exhibited higher basal activity of G_i_ signaling compared to other chemokine receptors such as CCR2, CCR5 and CCR10 (ref. ^25^).

### Recognition of CCR1 by CCL15

**Extended Data Fig. 7:**
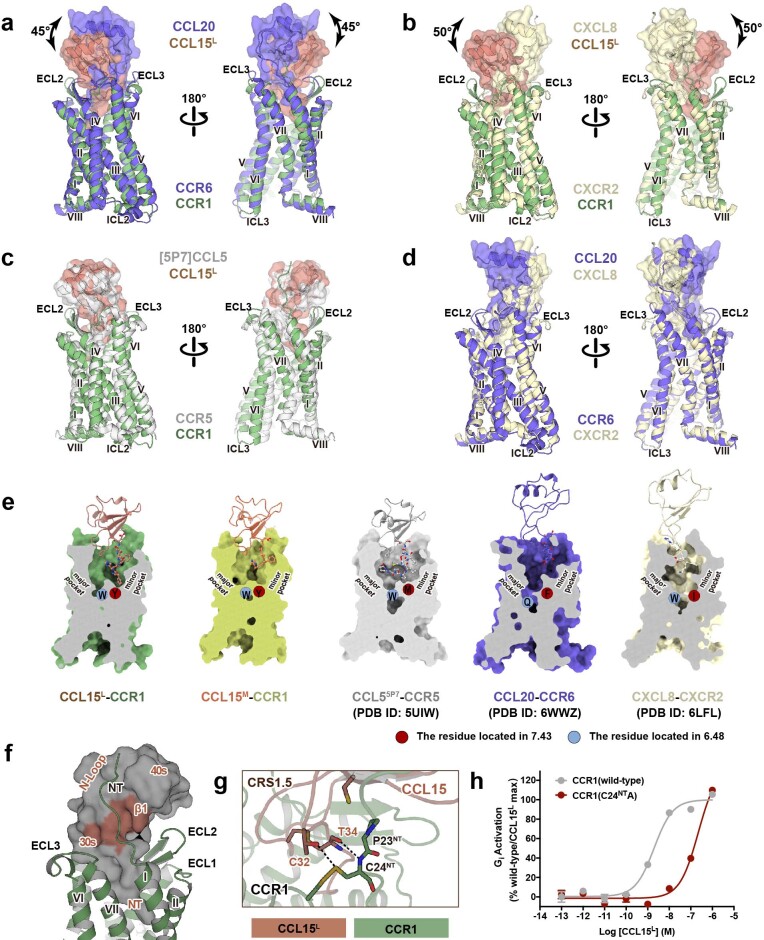
Comparison of apo, CCL15^M^-, and CCL15^L^-bound CCR1. **a-d**, Different orientations of chemokines (shown as surface) relative to the corresponding receptors (shown as ribbon). CCL15^L^ (brown)-CCR1 (green) vs. CCL20-CCR6 (purple) complex (**a**); CCL15^L^ (brown)-CCR1(green) vs. CXCL8-CXCR2 (yellow) (**b**); CCL15^L^ (brown)-CCR1 (green) vs. CCL5^5P7^-CCR5 (grey) (**c**); CCL20-CCR6 (purple) vs. CXCL8-CXCR2 (yellow) (**d**). **e**, Surface cut-away views of chemokine-binding pockets. Receptors were shown as surface, chemokines were shown as ribbon with the N-terminus shown as ball and sticks. CCL20-CCR6 (PDB ID: 6WWZ); CXCL8-CXCR2 (PDB ID: 6LFL); CCL5^5P7^-CCR5 (PDB ID: 5UIW). **f**, The CRS1.5 binding surface of CCL15^L^ with CCR1. The CRS1.5 region was highlighted in brown. **g**, Details of interactions between CCR1 and CCL15^L^ at the CRS1.5. Hydrogen bonds were depicted as dash lines. **h**, Effects of C24^NT^A mutation on G_i_ activation. N= three independent experiments, performed with single replicates. Data were shown as mean±SEM. Source data

The recognition of a ligand by chemokine receptors is a complex process that is still not fully understood^26–28^. Since the structural alignment of CCL15^L^- and CCL15^M^-CCR1 complexes revealed almost the same binding mode between the ligand and receptor, we used the structure of CCL15^L^–CCR1 complex as a model for clarity of presentation to explore the mode of CCL15 binding to CCR1 (Extended Data Fig. 6). So far, only a few high-resolution structures of chemokine receptors in complex with chemokines have been resolved^29–33^. As shown in Extended Data Fig. 7a–d, the orientation of the globular core of CCL15^L^ to CCR1 was similar to that of CCL5^5P7^ bound to CCR5 (Protein Data Bank (PDB) ID 5UIW), but rotated by about 50° when compared to the corresponding chemokines in the CCL20–CCR6 (PDB ID 6WWZ) and CXCL8–CXCR2 (PDB ID 6LFL) complexes, suggesting that diverse recognition modes exist among these chemokine–receptor interactions^29–31^.

**Fig. 3:**
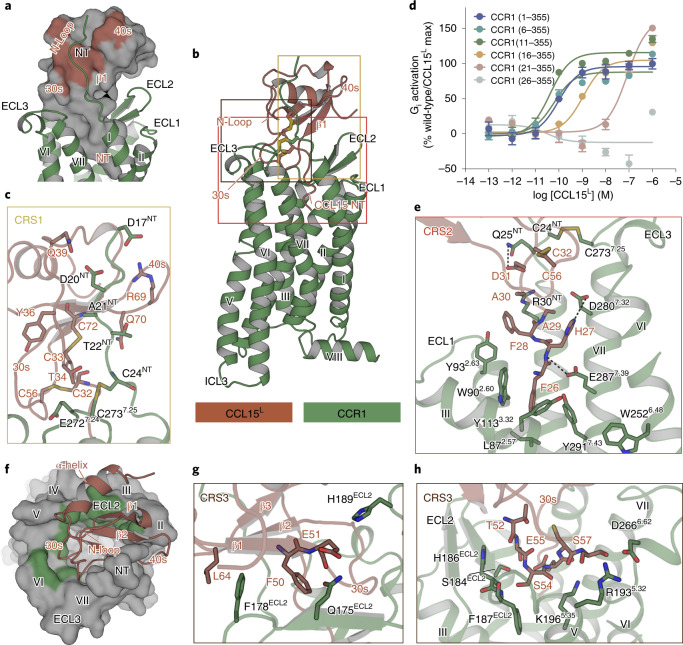
The recognition of CCR1 by CCL15. **a**, The CRS1 binding surface of CCL15^L^ with CCR1, in which the N terminus of CCR1 interacted with the N-loop of CCL15^L^. CCR1 is shown as a ribbon diagram in green, CCL15^L^ is shown as a gray surface and the interaction areas are highlighted in brown. **b**, Side view of the structure of CCL15^L^–CCR1 complex. **c**, The CRS1 binding surface of CCL15^L^ with CCR1. **d**, The dose–response curves of G_i1_ signaling in NanoBiT G protein dissociation assays of full-length and N-terminal sequential truncations of CCR1. Data are shown as mean ± s.e.m. *N* = eight independent experiments, performed with single replicates. **e**, The CRS2 binding surface of CCL15^L^ with CCR1. **f**, The CRS3 binding surface of CCL15^L^ with CCR1, in which the 30 s loop and β1 strand of CCL15^L^ interacted with the extracellular region of CCR1. CCL15^L^ is shown as a ribbon diagram in brown, CCR1 is shown as a gray surface and the interaction areas are highlighted in green. **g**,**h**, Details of interactions between CCR1 and CCL15^L^ at the CRS3 in different views. Hydrogen bonds are depicted as dashed lines. Source data

According to the classic ‘two-site’ model, the binding of chemokines to receptors involves two main interaction sites: (1) the chemokine recognition site 1 (CRS1), where the N terminus of the receptor interacts with the globular core of the chemokine and (2) the chemokine recognition site 2 (CRS2), where the N terminus of the chemokine interacts with the transmembrane binding pocket of the receptor^32,34^. The CCL15^L^-bound CCR1 structure shows that the CCL15^L^ is stably anchored in the extracellular half of the receptor 7TM domain and inserts deeply into the helical bundle (Fig. 3a). At the CRS1, the N terminus of CCR1 (residue D^17^YGDATPCQK^26^) ran parallel with the N-loop region of CCL15^L^ and fitted onto the groove formed by the N-loop and β3 strands of CCL15^L^, corresponding to a surface buried area of 745 Å^2^ (740.4 Å^2^ in CCL15^M^–CCR1) (Fig. 3a–c and Supplementary Table 3). Although the electron microscopy (EM) maps of both CCL15^L^–CCR1 and CCL15^M^–CCR1 only revealed the resolved density starting from the residue D17^NT^ in the N terminus of CCR1, the various N-terminal truncations of the first 20 residues of CCR1 retained partial activities of CCL15-induced G protein activation as determined by NanoBiT assay. Furthermore, the deletion of the first 25 residues of CCR1 almost eliminated CCL15-induced receptor activity, suggesting that the CCR1 N terminus plays a critical role in ligand recognition and receptor activation (Fig. 3d). At the CRS2, the N terminus of CCL15^L^ inserted into the pocket within the 7TM domain in a position that was notably deeper than other chemokines bound to the corresponding chemokine receptors (Extended Data Fig. 7e). As shown in Fig. 3e, the most N-terminal residue of CCL15^L^, F26, faced toward the minor pocket formed by L87^2.57^, W90^2.60^, Y113^3.32^ and Y291^7.43^. The backbone amide of H27 formed a hydrogen bond with E287^7.39^ (superscripts indicate the Ballesteros–Weinstein numbering scheme)^35^, consistent with the fact that the critical role of residue E287^7.39^, which is found in 74% of chemokine receptors and is crucial for chemokine-induced activity in many chemokine receptors^36,37^. Meanwhile, mutations of these F26-binding residues were previously reported to decrease the inhibitory effect of CCR1-targed small-molecule antagonists, suggesting that they were also important for activity of the antagonists^38,39^. It was also noteworthy that there were two side chain to side chain hydrogen bonds formed between H27 and D280^7.32^, D31 and Q25^NT^, respectively (Fig. 3e).

Besides the two classical chemokine recognition sites as discussed above, CCL5^5P7^ was previously reported interacting with CCR5 through an epitope designated as the CRS1.5, where the ^19^PC^20^ motif of CCR5 packed against the conserved disulfide of its ligand^29^. CRS1.5 was also observed in the CCL15^L^–CCR1 complex, with hydrogen bonds forming between C24^NT^ and CCL15 (Extended Data Fig. 7g). In agreement with that, C24^NT^A substitution significantly impaired the activation of the receptor (Extended Data Fig. 7h). Furthermore, additional interactions were observed between CCL15^L^ and CCR1, where β1-β2 strands and the 30s loop (residue ^50^FETSSECS^57^) of CCL15^L^ were found to interact with ECLs 2–3, and with TMs 5–6 of CCR1 (Fig. 3f–h). This recognition site was also observed in the CCL5^[5P7]^–CCR5 complex, which was designated as the CRS3 hereafter. The buried surface areas of the CRS3 of CCR1 (700 Å^2^ in CCL15^L^–CCR1 and 748.2 Å^2^ in CCL15^M^–CCR1, respectively) were comparable to the corresponding area in the CCL5^5P7^–CCR5 complex (698.5 Å^2^), far more significant than that in other chemokine receptor structures including the CXCL8–CXCR2 (300 Å^2^) and CCL20-CCR6 (448 Å^2^) complexes (Supplementary Table 3). The CCL15^L^ β1 strand ran antiparallel with ECL2 of CCR1 where these two parts interacted with each other primarily through hydrophobic effects. Meanwhile, the 30s loop of CCL15^L^ submerged into the 7TM pocket of CCR1, where it made contact with ECL2 and ECL3, and with TM5 and TM6 through comprehensive hydrogen bonds and hydrophobic interactions (Fig. 3g,h). Sequence alignment showed that the β1–β2 strands and the 30s loop were relatively more conserved than the N-loop among the endogenous ligands of CCR1, indicating the CRS3 might be a major determinant for ligand binding of CCR1 (Extended Data Fig. 1a).

### Y^7.43^ acted as a ‘toggle switch’ for biased signaling

**Fig. 4:**
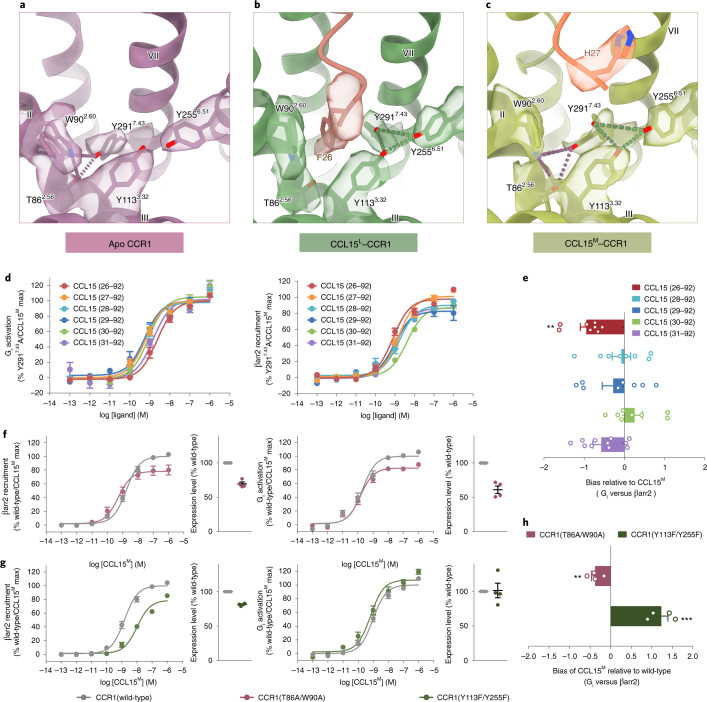
Structural basis of signal bias in CCR1. **a**–**c**, Close-up view of the CCL15-binding pocket of CCR1s in apo (**a**), CCL15^L^-bound (**b**) and CCL15^M^-bound (**c**) states. The EM density of F26 (**b**) and H27 (**c**) of CCL15, and residues T86^2.56^, W90^2.60^, Y113^3.32^, Y255^6.51^ and Y291^7.43^ of CCR1 are shown. Hydrogen bonds were depicted as dash lines. **d**, The effects of CCL15 truncations on CCR1(Y291^7.43^A). Dose–response curves for CCL15-induced β-arrestin2 recruitment (left) and G_i1_ signaling (right) measured by NanoBiT assay. *N* = eight independent experiments, performed with single replicates. **e**, Bias factors of CCL15 truncations relative to CCL15^M^. Bias factors derived from curve fit parameters from **d**. *N* = eight independent experiments, performed with single replicates. The asterisk symbols indicate statistically significant difference (*P* = 0.0067, *P* = 0.9986, *P* = 0.7725, *P* < 0.8323 and *P* = 0.1613 from top to bottom, **P < 0.01) for CCL15 truncations versus CCL15 (27–92), determined by one-way ANOVA. **f**,**g**, The effects of CCR1(T86^2.56^A/W90^2.60^A) (**f**) and CCR1(Y113^3.32^F/Y255^6.51^F) (**g**) on CCL15^M^-induced activation. *N* = four independent experiments, performed with quadruple replicates. **h**, Bias signaling induced by CCL15^M^ on CCR1(T86^2.56^A/W90^2.60^A) and CCR1(Y113^3.32^F/Y255^6.51^F) relative to CCR1(wild-type). Bias factors derived from curve fit parameters from **d**. *N* = four independent experiments, performed with quadruple replicates. *P* values were calculated using a two-tailed Student’s *t*-test. The asterisk symbols indicate statistically significant difference (*P* = 0.042 and 0.0003, from top to bottom, **P < 0.01, ***P < 0.001). In **d**–**h**, all data are shown as mean ± s.e.m. Source data

**Extended Data Fig. 8:**
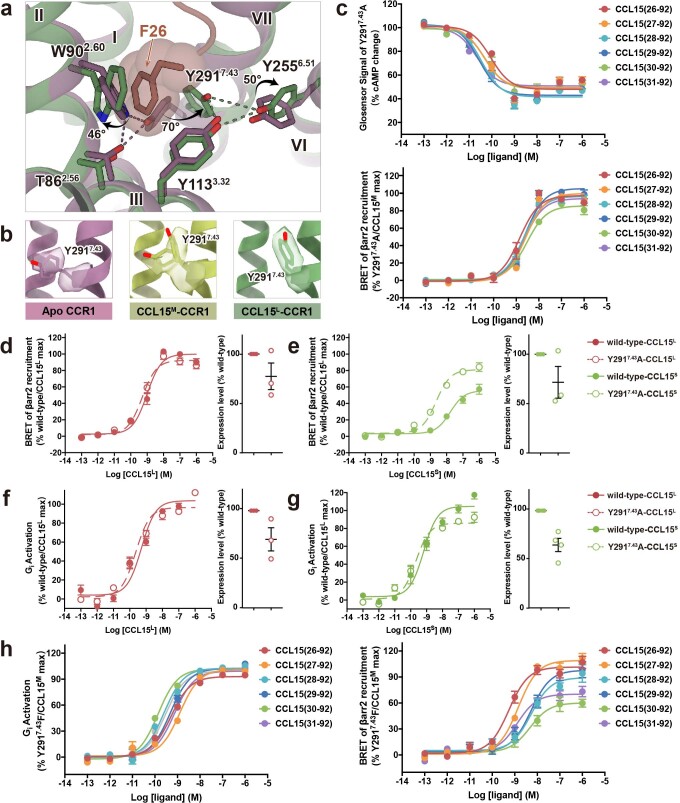
Molecular dynamics simulations. **a**, The orthosteric binding pocket of CCL15^L^-bound and apo CCR1. The hydrogen bonds were depicted as dash lines. **b**, EM density of residue Y291^7.43^ in CCL15^L^-bound (left), CCL15^M^-bound (middle) and apo state (right) CCR1. **c**, CCL15 truncations-induced G_i1_ (upper) and β-arrestin2 (bottom) signaling on CCR1 (Y291^7.43^A), measured by Glosensor and BRET assays, respectively. N= six (upper) or eight (bottom) independent experiments, performed with single replicates. **d-g**, The β-arrestin2 (**d-e**) and G_i_ signaling (**f-g**) induced by CCL15^L^ (**d, f**) and CCL15^S^ [CCL15(30-92)] (**e, g**). N= three (**d-e**) or four (**g**) independent experiments, performed with quadruple replicates. **h**, CCL15 truncations-induced G_i1_ (left) and β-arrestin2 (right) signaling on CCR1 (Y291^7.43^F), measured by NanoBiT and BRET assays, respectively. N= four (left) or eight (right) independent experiments, performed with single replicates. In **c-h**, all data were shown as mean±SEM. Source data

It is widely accepted that interactions between biased agonists and GPCRs result in the stabilization of a unique conformation adopted by the receptor that preferentially activates one downstream signaling^2^. Structural comparison of the extracellular pocket core of CCR1 among CCL15^L^-, CCL15^M^-bound and apo states was performed for the study of biased signaling. In the extracellular pocket core of apo CCR1, the side chain of Y291^7.43^ pointed toward TM2, forming hydrogen bonds with T86^2.56^ and W90^2.60^ (Fig. 4a). On the binding of the CCL15^L^ into the 7TM pocket of CCR1, the aromatic group of CCL15 F26 formed strong interactions with W90^2.60^ and Y291^7.43^. Hence, the resulting steric hindrance pushed the side chain of W90^2.60^ and Y291^7.43^ to sway from their original positions by 46° and 70°, respectively, thereby breaking the Y291^7.43^–W90^2.60^–T86^2.56^ polar network (Extended Data Fig. 8a). Consequently, the released Y291^7.43^ then formed hydrogen bonds with Y113^3.32^ and Y255^6.51^, establishing CCR1 in a conformation favorable for β-arrestin recruitment without strongly influencing G protein signaling (Figs. 1e and 4b). The density of Y^7.43^ in the CCL15^M^–CCR1 complex displayed two alternative conformations, with one conformation resembling the CCL15^L^-bound state and the other resembling the apo CCR1 state (Fig. 4c and Extended Data Fig. 8b). These findings illustrated that the CCL15^M^-induced conformation of Y^7.43^ was flexible and therefore retained partial capability of β-arrestin recruitment, which accounted reasonably well for the functional results (Fig. 1c,d and Extended Data Fig. 2c).

**Extended Data Fig. 9:**
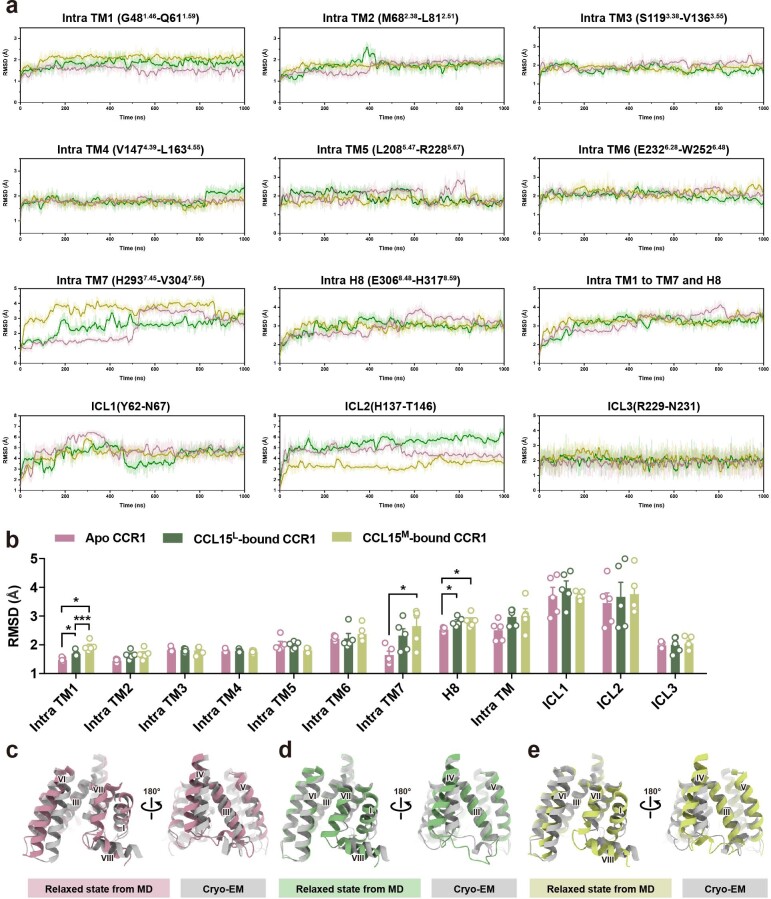
Effects of mutations in biased signaling. **a**, Representative RMSD analysis of all cytoplasmic part TMs, H8 and ICL region (apo CCR1, plum; CCL15^L^-bound CCR1, green; CCL15^M^-bound, dark khaki). **b**, Statistical analysis preformed on RMSD values. Error bars represented mean and SEM from five replicates. The asterisk symbols indicated statistically significant difference (*P <0.05, ***P <0.001) among CCR1 in different states by two-way ANOVA. **c-e**, Relaxed state of apo (**c**, plum), CCL15^L^-bound (**d**, green), and CCL15^L^-bound (**e**, dark khaki) CCR1s from 1us simulation snapshots with their cryo-EM structures (dark grey), respectively. Source data

Consistently, the variance of β-arrestin recruitment among all CCL15 N-terminal truncations was significantly decreased with alanine substitution of Y291^7.43^ in CCR1. By contrast, the effect of Y291^7.43^A mutation on G protein activation was relatively smaller, resulting in a strong decline in signaling bias of most CCL15^S^ truncations compared to CCL15^M^ (Fig. 4d,e, Extended Data Fig. 8c and Supplementary Table 5). As shown in Extended Data Fig. 8d,e, a Y291^7.43^A mutation significantly enhanced the CCL15^S^-induced β-arrestin recruitment pathway of CCR1, but hardly influenced either the CCL15^S^-induced G protein activation or any downstream signaling induced by CCL15^L^. A phenylalanine mutation of Y291^7.43^ in CCR1 was observed to be not strong enough to destroy the biased signaling induced by CCL15^S^ (Extended Data Fig. 8f). Taken together, these results indicate that it was the hydrophobic interaction between F26 and Y291^7.43^ that triggered the conformational change of Y291^7.43^, playing a critical role in β-arrestin recruitment. The side chain of Y291^7.43^ seemed to be unfavorable for β-arrestin recruitment in the presence of CCL15^S^. Moreover, molecular dynamics simulations of CCR1 with the protein removed showed that three distinctive structural configurations in the orthosteric pocket induced different conformational dynamics at the cytoplasmic half of the receptor, especially at the TM1, TM7 and H8 regions. These results indicate the differential conformational dynamics in response to the extracellular polar network exchange, which might contribute to the natural biased agonism of CCR1 (Extended Data Fig. 9).

**Extended Data Fig. 10:**
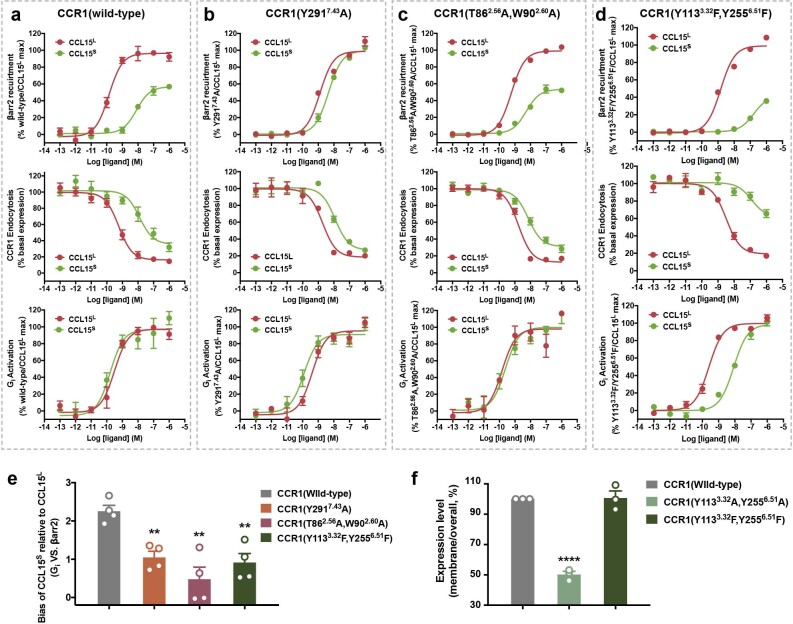
Effects of mutations in biased signaling. **a-d**, Dose-response curves of β-arrestin recruitment (upper), endocytosis (middle) and G_i_ activation (bottom) on CCR1(wild-type) (**a**), CCR1(Y291^7.43^A) (**b**), CCR1(T86^2.56^A/W90^2.60^A) (**c**) and CCR1(Y113^3.32^F/Y255^6.51^F) (**d**) induced by CCL15^L^ and CCL15^S^ [CCL15(30-92)]. N= three independent experiments for the endocytosis on CCR1(Y291^7.43^A) and CCR1(Y113^3.32^F/Y255^6.51^F), performed with single replicates. N= four independent experiments for the others, performed with single replicates. **e**, Biased factors of CCL15^S^ [CCL15(30-92)] on wild-type and mutational CCR1s relative to CCL15^L^. N= four independent experiments, performed with single replicates. The asterisk symbols indicated statistically significant difference (P =0.0072, P =0.0004, and P=0.0034 from left to right, **P <0.01) for mutants vs. wild-type CCR1 as determined by one-way ANOVA. **f**, The proportion of membrane to total expression of receptors. N= three independent experiments, performed with single replicates. P values were calculated using two-tailed Student’s t-test. The asterisk symbols indicated statistically significant difference (P <0.001, and P=0.9789 from left to right, ****P <0.0001) for mutants vs. wild-type CCR1. In **a-f**, all data were shown as mean±SEM. Source data

Furthermore, mutations of the complementary residues in the polar networks including the T86^2.56^A/W90^2.60^A and the Y113^3.32^F/Y255^6.51^F mutations, were also observed to reduce the difference of signal bias between CCL15^S^ and CCL15^L^, suggesting that the residues within this region were important for signal transduction of CCR1 (Extended Data Fig. 10a–e). It should be mentioned that the mutation of Y113^3.32^A/Y255^6.51^A lead to an almost complete loss of CCR1 expression localized on plasma membrane, suggesting that the residues of Y113^3.32^ and Y255^6.51^ participated in maintaining the three-dimensional (3D) structure of CCR1 (Extended Data Fig. 10f). The T86^2.56^A/W90^2.60^A mutant displayed an increased signaling bias toward β-arrestin pathway, while the Y113^3.32^F/Y255^6.51^F mutation increased the bias toward G protein signaling (Fig. 4f-h and Supplementary Table 6). Taken together, these results demonstrated that distinct rotamers of Y291^7.43^ in CCR1 served as a sensor to discriminate different forms of CCL15 truncations, and mutations that disturbed the equilibrium balance of the intracellular polar network of Y291^7.43^ triggered biased signaling of CCR1.

## Discussion

Innate immune cells express multiple chemokine receptors and chemokines to control their migration, activation, differentiation and survival. At a cellular level, chemokines can synergize or antagonize with each other for downstream signal transduction. For instance, chemokine receptors not only could be stimulated by chemokines for G protein recruitments, but also undergo internalization on some chemokine agonist binding, leading to impairment of other chemokines’ efficacies. Therefore, molecular mechanisms of chemokine regulation and signaling bias of chemokine receptors are required for understanding of their functional intricacies.

In this paper, we identified and characterized the biased signaling properties of different CCL15 variants and their binding to CCR1. While the longer form of CCL15 (CCL15^L^) displayed β-arrestin-biased signaling, the shorter forms of CCL15 (CCL15^S^) showed gradual biased agonism toward G_i_-mediated signaling (Fig. 1). The cryo-EM structures we reported here complemented the molecular mechanisms of immunomodulation induced by these endogenous chemokines. Structural alignments revealed that CCL15 resembled an orientation similar to CCL5^[5P7]^–CCR5 (PDB ID 5UIW), but rotated by about 50° when compared to other chemokine–receptor complexes. This phenomenon suggested that at least two distinct binding modes existed in the chemokine recognition system (Extended Data Fig. 7a–d). In addition, both the CCL15–CCR1 and CCL5^[5P7]^–CCR5 complexes showed a new recognition site (the CRS3), where the 30s loop of the ligand was submerged into the 7TM pocket of its receptor, demonstrating it is a major determinant for ligand binding of CCR1 and CCR3.

Structural comparisons and functional experiments confirmed that conformational rearrangement of Y^7.43^ and exchange of polar networks in the orthosteric binding pocket of CCR1 lead to strongly biased signaling. In particular, the longer form of CCL15 (CCL15^L^), which has a significant interaction with Y^7.43^, displayed strong activation of both G_i_ protein and β-arrestin pathways. The shorter forms of CCL15 forms (CCL15^S^) have impaired ability to activate the β-arrestin pathway but not G_i_-mediated signaling, making them endogenous biased agonists prone to stimulating the G protein pathway. Y^7.43^ is conserved over 90 class A GPCRs, and 65 class A GPCRs have at least one polar residue at both polar network sides, including the μOR, κOR and AT_1_R (Supplementary Table 7). In two recent studies on the AT_1_R, Y^7.43^ was also found to be important for the allosteric regulation of receptor conformations in response to ligands with different bias profiles^4,40^. Unlike our observation in CCR1, Y^7.43^ in AT_1_R was proposed to be critical for ligand-dependent coupling of Gq signaling rather than β-arrestin coupling. Although the ultimate response to the movement of Y^7.43^ in CCR1 and AT_1_R was different, Y^7.43^ in both receptors was involved in determining the conformation of the intracellular half of TM7, and played a critical role in modulating downstream signaling. The side chain of Y^7.43^ in both CCL15^M^–CCR1 and AngII-AT_1_R complexes displayed highly dynamic changes, which seemed to be typical for more balanced ligands.

Together, our results provide structural insights into chemokine recognition, biased signaling and pharmacology of CCR1. Although CCR1 has been regarded as a candidate target for autoimmune and allergic diseases for a long time, the success of CCR1-targeted drug development has been limited. Manipulating the conformational exchange of the polar networks in the receptor orthosteric binding pocket to achieve bias signaling could be a general framework for the GPCRs with the conserved Y^7.43^, allowing rational design of biased ligands with an ultimate aim to achieve function-selective therapeutics with fewer side effects.

## Methods

### Expression and purification of CCL15 N-terminal truncations

The full-length of human CCL15 (1–92) complementary DNA was bought from Miaolingbio. The sequence of CCL15 (26–92) was cloned into a modified pFastBac1 vector, containing a GP67 signal peptide at the N terminus before the ligand to facilitate protein secretion. A maltose-binding protein (MBP) tag followed by a C-terminal 8*His tag was fused into the C terminal of CCL15 (26–92) with a linker containing a 3C protease cleavage site (LEVLFQGP). Using the bac-to-bac system, CCL15 (26–92)-3C-MBP-8*His was overexpressed by High Five insect cells. Insect cell cultures were grown in protein-free insect cell culture medium (Expression Systems ESF 921). After 48 h, the culture medium was collected and the initial purification was performed using Ni-NTA affinity chromatography (GE Healthcare). CCL15 (26–92)-3C-MBP-8*His was eluted with high imidazole elution buffer (20 mM HEPES pH 7.5, 100 mM NaCl and 250 mM imidazole). Then, the removal of C-terminal MBP and 8*His tag were achieved by 3C protease digestion (1:100). Next, 10% (w/v) glycerol was added together with 3C protease. Finally, purification to homogeneity of CCL15 (26–92) was achieved by size exclusion chromatography on a SuperdexTM 75 Increase 10/300 GL column (GE Healthcare) in size exclusion chromatography buffer (20 mM HEPES pH 7.5, 100 mM NaCl and 10% (w/v) glycerol), with the separation of CCL15 (26–92), 3C protease and MPB. About 1 mg of target proteins could be obtained from 1 l of culture medium. The other N-terminal-truncated CCL15 analogs were obtained by following a similar strategy to that described above, with the sequence of CCL15 (26–92) replaced by other truncations in the construction of recombinant plasmids.

### GloSensor cAMP assay

We fused a flag-tag into the N terminal of full-length CCR1, and cloned into pcDNA3.1 plasmids. Human embryonic kidney 293T (HEK293T) cells were transfected with a plasmid mixture consisting of pcDNA3.1-flag-CCR1 and the cAMP biosensor GloSensor-22F (Promega) at a ratio of 2:1. After 24 h, transfected cells were plated onto a 96-well plate, which was treated with cell adherent reagent (Applygen) in advance. After another 12 h, cells were treated with Hank’s balanced salt solution for starvation and then incubated in CO_2_-independent media containing 2% GloSensor cAMP Reagent (Promega) at a volume of 50 μl per well. Then 1 μM Forskolin (5.5 μl) (Sigma) was added to each well and incubated for 20 min at room temperature before measurements for baseline luminescence (Spark Multimode microplate reader, TECAN). Next, test ligands (CCL15 variants, 6 μl) were added at different concentrations from 10^−6^ to 10^−13^ M. All luminescence values were first normalized by the initial counts before ligand treatments. Fold-change signals over the treatment of the lowest CCL15 concentration were used to show intracellular cAMP response. We carried out nonlinear regression analysis using a sigmoidal dose response in GraphPad Prism to calculate the values of *E*_max_ and half-maximum effective concentration (EC_50_).

### Bioluminescence resonance energy transfer (BRET) assay

β-arrestin recruitment was measured by a BRET assay. The Rluc8 fragment was inserted into the C terminal of CCR1 (1–327 aa) with a linker of 6*His. The Venus fragment was inserted into the N terminal of β-arrestin2. These sequences were then cloned into pBiT1.1 plasmids (Promega). HEK293T cells were transiently transfected with CCR1-Rluc8 and Venus-β-arrestin2 (1:1). The transfected cells were seeded onto a cell adherent reagent (Applygen)-coated plate (Corning). After 48 h, cells were washed once and maintained in the buffer (5 mM HEPES pH 7.4 and 0.01% BSA) at a volume of 20 µl per well. Then, the transfected cells were stimulated with ligands (5 µl) at different concentrations from (10^−6^ to 10^−13^ M) for 40 min. After the addition of luciferase substrate coelenterazine h (5 µM), the BRET signals were determined as the ratio of light emitted by Venus-tagged biosensors and light emitted by Rluc8-tagged biosensors. The BRET acceptor (520–560 nm) and BRET donor (460–485 nm) emission signals were measured using the Spark Multimode microplate reader (Tecan). Venus fluorescence was measured before reading luminescence and calculated as average fluorescence from each control well. The BRET signal from the same well was determined as the ratio of the light emitted by Venus (520–560 nm) over that emitted by RLuc8 (460–485 nm). We carried out nonlinear regression analysis using a sigmoidal dose response in GraphPad Prism to calculate the values of *E*_max_ and EC_50_.

### Flow cytometry-based endocytosis assays

Human monocytic THP-1 cells (ATCC-TIB-202) were cultured in RPMI 1640 (Hyclone) supplemented with 10% FBS (Invitrogen) and antibiotics (Sangon). First, cells were plated on to a 96-well plate (Corning) and incubated with CCL15 truncations for 2 h at 37 °C. Then the THP-1 cells were counterstained with allophycocyanin (APC) anti-CCR1 (Biolegend, used in 1:1,000 dilution) and 4,6-diamidino-2-phenylindole (DAPI) (C_16_H_15_N_5_·2HCl, Sigma, used in 1:10,000 dilution). DAPI (Thermo Fisher) staining was used to exclude dead cells. With the using of CytoFlex (Beckman CytoFlex), gating using Fourier shell correlation- (FSC-)A versus SSC-A was performed to exclude cell debris. FSC-H versus FSC-A was used to distinguish single cells. The endocytosis of CCR1 mutations was investigated by the use of HEK293T cells. So that the amount of CCR1 expression on infected HEK293T cells could be similar to the amount of endogenous CCR1 expressed on monocytes, we used the pBiT1.1 vector (Promega) with the HSV-TK promoter, which provided constitutive, low-level expression in mammalian cells. The recombined vector used in this assay also contained an enhanced green fluorescent protein reporter downstream of the receptor and separated by a P2A self-cleaving peptide (ATNFSLLKQAGDVEENPGP). The transfected cells were incubated with corresponding CCL15 truncations for 2 h, and then stained with Phycoerythrin (PE) anti-DYKDDDDK Tag Antibody (Biolegend #637310, used in 1:1,000 dilution) and DAPI antibody (Sigma). The gate strategy was similar to that used in THP-1 cells as described above, except that the successful transfected HEK293T cells were further identified with high fluorescein isothiocyanate fluorescence.

Data were acquired on a CytoFlex Cytometer and analyzed with CytExpert software. For THP-1 cells, the endocytosis level was measured by calculating the ratio of the median APC fluorescence intensity between THP-1 cells treated with ligand in a test and lowest concentration (10^−13^ M). For HEK293T cells, the median PE fluorescence intensity was first normalized by the fluorescence intensity of fluorescein isothiocyanate, then further divided by the corresponding value of HEK293T cells treated with ligand in the lowest concentration (10^−13^ M). All data were normalized to 100% for presentation. We carried out nonlinear regression analysis using a sigmoidal dose response in GraphPad Prism to calculate the values of *E*_max_ and EC_50_.

### Purification of scFv16

The expression and purification of scFv16 were achieved as previously described^41^. In brief, the scFv16 was overexpressed and secreted into the culture medium of transfected High Five cells. Following affinity chromatography on Ni-NTA, the elution was purified by size exclusion chromatography on a Superdex 200 Increase 10/300 GL column (GE Healthcare). Then the monomeric fractions were concentrated, flash frozen and stored at −80 °C until use.

### Expression and purification of CCL15^L^–CCR1-G_i_, CCL15^M^–CCR1-G_i_ and apo CCR1–G_i_ complexes

For the purification of CCL15^L^–CCR1–G_i_, the full-length cDNA sequence of wild-type human CCR1 was fused with a LgBiT subunit (Promega) at the C terminus followed by a double MBP tag via a GS linker containing a 3C protease cleavage site. The sequence of CCL15 (26–92) was fused into the N terminus of CCR1 via a GS linker containing a tobacco etch virus (TEV) protease cleavage site. A dominant-negative Gα_i1_ (DNGα_i1_) was generated by site-directed mutagenesis to decrease the affinity of nucleotide-binding, and the Gβ1 was fused with a C-terminal SmBiT (peptide 86, Promega). Using the bac-to-bac system (Invitrogen), the virus of CCL15 (26–92)–CCR1-LgBiT-2*MBP was infected with the ones of Gα_i1_ and Gβγ-SmBiT at equal multiplicities of infection in SF9 insect cells. After 48 h of expression, the infected SF9 cells were resuspended in 20 mM HEPES pH 7.5, 2 mM MgCl_2_, 100 mM NaCl and protease inhibitor cocktail. The membranes were then solubilized with the addition of 0.5% (w/v) lauryl maltose neopentyl glycol and 0.1% (w/v) cholesterol hemisuccinate. Following incubation with Amylose resin (NEB), the protein was eluted with 10 mM maltose and treated with the TEV protease treatment to break the linker between chemokine and receptor, the 3C protease for the removal of 2*MBP protein, as well as the antibody scFv16 for complex stabilization. Finally, the purification of CCL15 (26–92)–CCR1–G_i_ complex to homogeneity was achieved by size exclusion chromatography on a Superose 6 Increase 10/300 GL column (GE Healthcare) in size exclusion chromatography buffer (20 mM HEPES pH 7.5, 100 mM NaCl, 0.00075% lauryl maltose neopentyl glycol, 0.0002% (w/v) cholesterol hemisuccinate and 0.00025% (w/v) glyco-diosgenin). The same strategy was used for the purification of CCL15 (27–92)–CCR1–G_i_ and apo CCR1–G_i_ complex, except that the sequence of CCL15 (26–92) was replaced by CCL15 (27–92) or CCL3 (4–69).

### N-terminal end sequencing based on mass spectrometry and sample preparation

The cDNA sequence of CCL15 (22–92) was fused into the N terminus of CCR1 via a GS linker containing a TEV protease cleavage site (ENLYFQS). Expression and purification of the CCL15 (22–92)–CCR1–G_i_ complex were performed by following a strategy similar to that used for obtaining CCL15^L^- and CL15^M^-bound CCR1 complexes as described above. Monomeric fractions were loaded on 12.5% SDS–PAGE and stained with Coomassie Blue. The gel band corresponding to the ligand-receptor chimera was cut, digested and subjected to liquid chromatography–tandem mass spectrometry analysis as described previously^42^. Briefly, the gel was destained in fixing buffer (50% (v/v) methanol, 5% (v/v) acetic acid in water). Following reduction and alkylation, the peptides were digested with trypsin. The peptides were extracted by using gradient acetonitrile and desalted by PierceTM C18 Spin Tips. The cleaned peptides were then separated using the Ultimate 3000 nanoliquid chromatography–tandem mass spectrometry system with a 30-min gradient and analyzed by QE-HFX (Thermo Fisher). The identification and quantification of sequences were analyzed by pFind (v.3.1.5). The search parameters were set as follows: carbamidomethylation of cysteine as the fixed modification, oxidation of methionine as the variable modification and trypsin as the digestion enzyme. The quantification results were ranked by spectra counts (propensity score matching <0.01).

### Cryo-EM grid preparation and data collection

The purified CCL15^L^-, CCL15^M^-bound or apo CCR1–G_i_ complexes (3 μl) were applied onto a glow-discharged holey carbon grid (Quantifoil R1.2/1.3) at roughly 5 mg ml^−1^. The Grids were plunge into liquid ethane using Vitrobot Mark IV (Thermo Fischer Scientific) and subsequently transferred to liquid nitrogen and stored for data collection. Cryo-EM imaging was performed on a Titan Krios at 300 kV using Gatan K2 Summit detector in the Center of Cryo-Electron Microscopy, Zhejiang University (Hangzhou, China). Micrographs were recorded in counting mode at a dose rate of about 8.0 e Å^2^ s^−1^ with a defocus ranging from −1.0 to −3.0 μm using SerialEM software^43^. The total exposure time was 8 s and 40 frames were recorded per micrograph. A total of 5792, 6,230 and 5,431 videos were collected for the CCL15^L^-, CCL15^M^-bound or apo CCR1–G_i_ complex, respectively.

### Image processing and map construction

Cryo-EM image stacks were aligned using MotionCor2 v.1.3.2 (ref. ^44^). Contrast transfer function (CTF) parameters were estimated by Gctf v.1.18 (ref. ^45^). Particle selections for two-dimensional (2D) and 3D classifications were performed on a binned dataset with a pixel size of 2.028 Å using RELION v.3.0.8.

For the CCL15^L^–CCR1–G_i_ complex, 5,812,667 particles yielded by automated particle picking were subjected to 2D classification to discard fuzzy subsets of particles, producing 5,507,136 particles. The map of the NTSR1–G_i_ complex (EMDB-20180) low-pass filtered to 60 Å was used as an initial reference model for two rounds of 3D classification, resulting in two well-defined subsets with 2,081,356 particles^46^. The selected subsets were subsequently subjected to 3D classification with a mask on the receptor. One subset shows the high-quality receptor density was selected, producing 1,090,180 particles. The selected subset was subsequently subjected to 3D refinement, CTF refinement, Bayesian polishing to reduce background noise and improve EM map quality. The final refinement generated a map with an indicated global resolution of 2.6 Å at a Fourier shell correlation of 0.143.

For the CCL15^M^–CCR1–G_i_ complex, 3,852,738 particles yielded by automated particle picking. Then particles were subjected to 2D and 3D classification to discard fuzzy subsets of particles, producing 1,336,807 particles. The map of the CCL15^L^–CCR1–G_i_ complex, low-pass filtered to 60 Å, was used as an initial reference model for the 3D classification, resulting in two well-defined subsets. The selected subsets were subsequently subjected to 3D classification with a mask on the receptor and ligand CCL15^M^. One subset shows the high-quality receptor density was selected, producing 423,872 particles. The selected subset was subsequently subjected to 3D refinement, CTF refinement, Bayesian polishing to reduce background noise and improve EM map quality. The final refinement generated a map with an indicated global resolution of 2.7 Å at a Fourier shell correlation of 0.143.

For the apo CCR1–G_i_ complex, 3,252,536 particles yielded by automated particle picking were subjected to 2D classification to discard fuzzy subsets of particles, producing 2,073,895 particles. The map of CCL15^M^–CCR1–G_i_ complex low-pass filtered to 40 Å was used as an initial reference model for a round of 3D classification, resulting in a well-defined subset with 965,870 particles. The selected subset was subsequently subjected to two rounds of 3D classification with a mask on the receptor and part of the G protein. Two subsets showing the high-quality density were selected, producing 391,181 particles. The selected particles were subsequently subjected to 3D refinement and the final refinement generated a map with an indicated global resolution of 2.9 Å at a Fourier shell correlation of 0.143. All local resolutions of these complexes were determined using the Bsoft package (v.2.0.7) with half maps as input maps^47^.

### Model building and refinement

For the structure of the CCL15^L^–CCR1–G_i_ complex, the initial model of CCR1 was downloaded from the predicted active CCR1 model from GPCRdb, and the initial CCL15 model was built from the NMR CCL15 structure (PDB ID 2HCC)^48^. The initial G_i_ and scFv16 complex was generated from the NTSR1–G_i_ complex (PDB ID 6OS9)^46^. Then, the CCL15^L^–bound CCR1-G_i_ model was used as the initial models of apo and CCL15^M^-bound CCR1–G_i_ complexes. The models were docked into the cryo-EM density map using chimera. After the initial docked models were refined using Rosetta, the models were subjected to iterative rounds of manual adjustment and auto refinement in Coot v.0.9.4 and Phenix v.1.18, respectively. The final refinement scores were validated by the module ‘comprehensive validation (cryo-EM)’ in Phenix. The model versus map FSC was used to analysis the fitting of the refined model to the cryo-EM map. The model versus two half maps FSC curves were also compared to avoid the overfitting of model toward the map. Structure figures were prepared by PyMOL v.2.5, Chimera v.1.15 and ChiemraX v.1.2.5.

### Ligand bias calculated by NanoBiT assays

The NanoBiT assay performed for the measurement of G protein activation was performed as previously described^46^. And for the measurement of ligand-induced β-arrestin recruitment, the LgBiT was inserted into the C terminal of CCR1 (1–327 amino acids (aa)), and the SmBiT was N terminally fused to β-arrestin2. These sequences were then cloned into pBiT1.1 plasmids (Promega) to achieve a low-level expression in mammalian cells. These two plasmids were transfected into HEK293T cells in equal proportions. After 24 h of incubation, the transfected cells were plated onto a plate (Corning), which was treated with cell adherent reagent (Applygen) in advance. After another 12 h of incubation, the transfected cells were washed once with Hank’s balanced salt solution buffer, and then maintained in the same buffer containing 5 mM HEPES pH 7.4, 0.01% BSA and 5 µM coelenterazine h (yeasen) at a volume of 25 μl per well. After incubation for 30 min, the plate was measured for baseline luminescence (Spark Multimode microplate reader, TECAN). Every ligand (5 μl) was added at different concentrations from (10^−6^ to 10^−13^ M) before second measurement. Luminescence counts were normalized to the initial count. Fold-change signals over the lowest concentration treatment of corresponding ligand were calculated. Finally, data were normalized to 100% of the maximal CCL15 (27–92) response for wild-type CCR1 using a sigmoidal dose response in GraphPad Prism.

The bias factors (β value) were determined by applying the following equation:

$$\beta \,{{{\mathrm{value}}}} = {{{\mathrm{log}}}}\left( {\left[ {\frac{{E_{{\mathrm{max}},P1}}}{{{\mathrm{EC}}_{50,P1}}}\frac{{{\mathrm{EC}}_{50,P2}}}{{{{E}}_{{\mathrm{max}},P2}}}} \right]_{{\mathrm{ligand}}} \times \left[ {\frac{{{{E}}_{{\mathrm{max}},P2}}}{{{\mathrm{EC}}_{50,P2}}}\frac{{{\mathrm{EC}}_{50,P1}}}{{{{E}}_{{\mathrm{max}},P1}}}} \right]_{{\mathrm{reference}}}} \right)$$

where *P*1 is NanoBiT G_i_ protein dissociation; *P*2, NanoBiT β-arrestin recruitments; a *β* value >0 denotes G_i_-protein biased and a *β* value <0 is β-arrestin biased.

Parameters used in this equation were based on the curve fits of the combined datasets described above.

### Molecular dynamics simulations

CCL15^L^-bound, CCL15^M^-bound and apo state of CCR1 models were substrate from the CCL15^L^–CCR1–G_i_, CCL15^M^–CCR1–G_i_ and apo CCR1–G_i_ complexes, respectively. The orientations of receptors are calculated by the Orientations of Proteins in Membranes database. Following that, the whole systems were prepared by the CHARM-GUI and embedded in a bilayer consisting of 200 1-palmitoyl-2-oleoyl-sn-glycero-3-phosphocholine lipids by replacement methods. The membrane systems were then solvated into a periodic TIP3P water box supplemented with 0.15 M NaCl. The CHARMM36m Force Field was used to model protein molecules. Then these systems were subjected to minimization for 10,000 steps using the conjugated gradient algorithm, and then heated and equilibrated at 310.13 K and 1 atm for 200 ps with 10.0 kcal mol^−1^ Å^−2^ harmonic restraints in the NAMD v.2.13. Then there were five cycles of equilibration for 2 ns each at 310.13 K and 1 atm, at which the harmonic restraints were 5.0, 2.5, 1.0, 0.5 and 0.1 kcal mol^−1^ Å^−2^ in sequence. Production simulations were run at 310.13 K and 1 atm in the NPT ensemble using the Langevin thermostat and Nose–Hoover method for 250 ns. Electrostatic interactions were calculated using the particle mesh Ewald method with a cutoff of 12 Å. Throughout the final stages of equilibration and production, 5.0 kcal mol^−1^ Å^−2^ harmonic restraints were placed on the residues of two state CCR1 models that were within 5 Å of ligand CCL15 include the five key residues (T86^2.56^, W90^2.60^, Y113^3.32^, Y255^6.51^, Y291^7.43^) related to biased signaling. Trajectories were visualized and analyzed using VMD v.1.9.3. The representative r.m.s.d. analysis presented in Extended Data Fig. 1a was performed by Origin2018, and statistical analysis of Extended Data Fig. 1b was performed by GraphPad Prism v.7.

### Flow cytometric analysis of the receptor expression

Flow cytometric analyses were performed by using CytoFlex (Beckman CytoFlex). The transfected cells were stained with PE anti-flag (Biolegend). Cells were gated by FSC-A versus SSC-A to exclude debris and then by FSC-H versus FSC-W to exclude cell doublets. Single cell APC fluorescence intensities were determined from over 3000 cells per experiment, which reflected the membrane protein expression level. All data of mutated CCR1 were normalized to the expression level of wild-type receptor in the same experiment. Values were shown as a percentage of the wild-type, which was set to 100%.

### Reporting Summary

Further information on research design is available in the Nature Research Reporting Summary linked to this article.

## Online content

Any methods, additional references, Nature Research reporting summaries, source data, extended data, supplementary information, acknowledgements, peer review information; details of author contributions and competing interests; and statements of data and code availability are available at 10.1038/s41589-021-00918-z.

## Supplementary information

Supplementary Information Supplementary Tables 1–7.

Reporting Summary

## Data Availability

Cryo-EM maps of apo CCR1–G_i,_ CCL15^M^–CCR1–G_i_ and CCL15^L^–CCR1–G_i_ complexes have been deposited in the Electron Microscopy Data Bank under accession codes EMD-32020, EMD-32021 and EMD-32022, respectively. The atomic coordinates of apo CCR1–G_i,_ CCL15^M^–CCR1–G_i_ and CCL15^L^–CCR1-G_i_ complexes have been deposited in the PDB under accession codes 7VL8, 7VL9 and 7VLA, respectively. All other data are available upon request to the corresponding authors. Publicly available datasets used in this study are: PDB IDs 5UIW, 6WWZ, 6LFL, 2HCC and 6OS9. Source data are provided with this paper.

## Source data

Source Data Fig. 1 Statistical source data.
Source Data Fig. 3 Statistical source data.
Source Data Fig. 4 Statistical source data.
Source Data Extended Data Fig. 2 Statistical source data.
Source Data Extended Data Fig. 3 Statistical source data.
Source Data Extended Data Fig. 7 Statistical source data.
Source Data Extended Data Fig. 8 Statistical source data.
Source Data Extended Data Fig. 9 Statistical source data.
Source Data Extended Data Fig. 10 Statistical source data.

## Extended data

are available for this paper at 10.1038/s41589-021-00918-z.
