# Supplementary material for: Identification and mechanism of G protein-biased ligands for chemokine receptor CCR1
Source: Nat Chem Biol. 2021 Dec 23;18(3):264–71. doi: 10.1038/s41589-021-00918-z (PMC8885419; doi:10.1038/s41589-021-00918-z)
Supplement: Supplementary file 1 — Supplementary Tables 1–7. [file 41589_2021_918_MOESM1_ESM.pdf]

nature portfolio

https://doi.org/10.1038/s41589-021-00918-z

Supplementary information

Identification and mechanism of G protein-biased ligands for chemokine receptor CCR1

In the format provided by the authors and unedited

Supplementary Tables

Supplementary Table 1 I Effects of CCL15 truncations on wild-type CCR1. The effects of CCL15 N-terminal truncations for G_i activation and β-arrestin2 recruitment were measured by NanoBiT assay and normalized to maximal CCR1 (wild-type)/ CCL15(27-92) response. Endocytosis results were calculated by dividing the final fluorescence intensity of sample tubes by the one of negative control. N= eight (G_i activation and β-arrestin2 recruitment) or six (endocytosis) independent experiments, performed with single replicates. Superscripts indicated statistically significant difference (****P<0.0001, ***P<0.001, **P<0.01, *P<0.05, and NS was no significance) for every CCL15 truncations vs. CCL15(27-92) as determined by one-way ANOVA. All data were shown as mean±SEM. The data were related to curves shown in **Fig. 1b-e**.

Ligand	G_i Activation		β-arrestin2 Recruitment		Bias Value (G_i VS. β-arrestin2)	n	Endocytosis		
	log EC50	Emax	log EC50	Emax			log EC50	Emax	n
**CCL15(26-92)**	-9.13±0.20^{NS}	105.9±4.4^{NS}	-9.61±0.13^{NS}	102.9±2.9^{NS}	-0.71±0.14*	8	-9.02±0.05^{NS}	78.9±2.7^{NS}	6
	(P=0.2418)	(P=0.9999)	(P=0.6011)	(P=0.9727)	(P=0.0115)		(P=0.6939)	(P=0.4696)	
**CCL15(27-92)**	-9.63±0.17	100.0±0.0	-9.38±0.08	100.0±0.0	0.00±0.00	8	-8.78±0.21	68.8±2.2^{NS}	6
**CCL15(28-92)**	-9.36±0.23^{NS}	98.9±3.8^{NS}	-8.77±0.07*	65.5±5.2****	0.40±0.22^{NS}	8	-7.22±0.08****	70.5±5.4^{NS}	6
	(P=0.7767)	(P=0.7006)	(P=0.0398)	(P< 0.0001)	(P=0.2682)		(P< 0.0001)	(P=0.9996)	
**CCL15(29-92)**	-9.49±0.22^{NS}	103.5±3.6^{NS}	-8.72±0.05**	73.1±4.6****	0.59±0.19*	8	-6.86±0.16****	74.7±4.9^{NS}	6
	(P=0.9793)	(P=0.9950)	(P=0.0095)	(P< 0.0001)	(P=0.0426)		(P< 0.0001)	(P=0.8661)	
**CCL15(30-92)**	-9.40±0.15^{NS}	101.8±6.0^{NS}	-8.18±0.14****	50.2±4.3****	1.29±0.15****	8	-7.22±0.08****	71.4±6.7^{NS}	6
	(P=0.8681)	(P=0.9494)	(P< 0.0001)	(P< 0.0001)	(P< 0.0001)		(P< 0.0001)	(P=0.9957)	
**CCL15(31-92)**	-9.69±0.15^{NS}	100.5±4.0^{NS}	-8.72±0.09**	55.0±2.6****	1.00±0.14***	8	-7.35±0.11****	65.8±5.8^{NS}	6
	(P=0.9996)	(P=0.8638)	(P=0.0022)	(P< 0.0001)	(P=0.0003)		(P< 0.0001)	(P=0.9897)	

Supplementary Table 2 l Cryo-EM data collection, model refinement and validation statistics.

	Apo-CCR1-G_i (EMDB-32020) (PDB 7VL8)	CCL15^M-CCR1-G_i (EMDB-32021) (PDB 7VL9)	CCL15^L-CCR1-G_i (EMDB-32022) (PDB 7VLA)
**Data collection and processing**			
Magnification	49,310	49,310	81,000
Voltage (kV)	300	300	300
Electron exposure (e–/Å^2)	62	62	70
Defocus range (μm)	-0.5 ~ -2.5	-0.5 ~ -2.5	-0.5 ~ -3.0
Pixel size (Å)	1.014	1.014	1.071
Symmetry imposed	Cl	Cl	Cl
Initial particle images (no.)	3,252,536	3,852,738	5,812,667
Final particle images (no.)	291,181	423,872	1,090,180
Map resolution (Å)	2.9	2.7	2.6
FSC threshold	0.143	0.143	0.143
Map resolution range (Å)	2.5~5.0	2.5~5.0	2.5~5.0
**Refinement**			
Initial model used (PDB code)	6OS9	6OS9	6OS9
Model resolution (Å)	3.4	2.8	2.7
FSC threshold	0.5	0.5	0.5
Model resolution range (Å)	3.4~5.0	2.8~5.0	2.7~5.0
Map sharpening *B* factor (Å^2)	-113.74	-78.8	-89.73
Model composition			
Non-hydrogen atoms	8834	9675	9605
Protein residues	1132	1222	1214
Ligands	59.14	32.73	39.97
B factors (Å^2)			
Protein	99.37	39.92	46.54
Ligand	59.14	32.73	39.97
R.m.s. deviations			
Bond lengths (Å)	0.008	0.005	0.007
Bond angles (°)	0.937	0.852	0.937
Validation			
MolProbity score	1.52	1.59	1.60
Clashscore	5.87	9.66	8.10
Poor rotamers (%)	0.00	0.09	0.00
Ramachandran plot			
Favored (%)	96.77	97.60	97.07
Allowed (%)	3.23	2.40	2.93
Disallowed (%)	0	0	0

Supplementary Table 3 | Binding area decomposition of different ligand-receptor complexes.

The structures of chemokine receptors included in this table were CCL5^{5P7}-CCR5 (PDB ID: 5UIW), CCL20-CCR6 (PDB ID: 6WWZ) and CXCL8-CXCR2 (PDB ID: 6LFL).

	CRS1	CRS2 (+CRS1.5)	30s loop(CRS3)	Total	CRS1+CRS2 (+CRS1.5)	State
**CCL15^{M}-CCR1**	740.4	549.5	748.2	1905	1268	Active
**CCL15^{L}-CCR1**	745.0	772.9	709.4	1954	1328	Active
**CCL5^{5P7}-CCR5**	339.5	712.9	698.5	1600	1009	Inactive
**CCL20-CCR6**	607.8	479.1	448.1	1414	1038	Active
**CXCL8-CXCR2**	823.3	667.0	300.0	1735	1489	Inactive

Supplementary Table 4 | Comparisons between effects of CCL15^{L} and CCL15^{S} on wild type CCR1 and other mutations.

NanoBiT results of G protein dissociation and β-arrestin recruitment were normalized to maximal response of CCL15^{L}[CCL15(26-92)]. The CCL15(30-92) was applied as the representative truncation of CCL15^{S} in the corresponding experiments. Endocytosis results were calculated by dividing the final fluorescence intensity of sample tubes by the one of negative control. N= three independent experiments for the endocytosis assay, performed with single replicates. N= three independent experiments for the endocytosis on CCR1(Y291$^{7.43}$A) and CCR1(Y113$^{3.32}$F/Y255$^{6.51}$F), performed with single replicates. N= four independent experiments for the others, performed with single replicates. The statistical difference between CCL15^{L}- and CCL15^{S}-induced signaling was calculated using two-tailed Student's t-test. And the statistical difference among mutants and wild-type CCR1 was determined by one-way ANOVA. Superscripts indicated statistically significant difference (****P<0.0001, ***P<0.001, **P<0.01, and NS was no significance). All data were shown as mean±SEM. The data were related to curves shown in **Extended Data Fig. 7c-g**.

		Wild-type			Y291$^{7.43}$A			T86$^{2.56}$A/W90$^{2.90}$A			Y113$^{3.32}$F/Y255$^{6.51}$F		
		CCL15^{L}	CCL15^{S}	n	CCL15^{L}	CCL15^{S}	n	CCL15^{L}	CCL15^{S}	n	CCL15^{L}	CCL15^{S}	n
β-arrestin2 Recruitment	log EC50	-9.89 ±0.16	-8.01 ±0.14*** P=0.0001	4	-8.89 ±0.07	-8.36 ±0.09** P=0.0035	4	-9.23 ±0.02	-8.28 ±0.03**** P< 0.0001	4	-8.83 ±0.03	-6.78 ±0.09**** P< 0.0001	4
	Emax	100.0 ±0.0	56.1 ±4.5**** P< 0.0001	4	100.0 ±0.0	99.6 ±4.3^{NS} P=0..9288	4	100.0 ±0.0	52.9 ±2.3**** P< 0.0001	4	100.0 ±0.0	41.7 ±2.7**** P< 0.0001	4
Endocytosis	log EC50	-9.27 ±0.14	-8.40 ±0.53	4	-8.72 ±0.08	-7.92 ±0.02	3	-8.78 ±0.12	-8.14 ±0.17	4	-8.48 ±0.07	-8.26 ±1.33	3
	Emax	83.9 ±3.7	64.9 ±5.8	4	81.1 ±6.4	74.0 ±3.6	3	87.5 ±1.3	67.8 ±3.6	4	81.0 ±3.7	35.5 ±3.9	3
G_i Activation	log EC50	-9.59 ±0.15	-9.78 ±0.04^{NS} P=0.2737	4	-9.38 ±0.07	-9.92 ±0.11** P=0.0056	4	-9.84 ±0.05	-9.77 ±0.37^{NS} P=0.8652	4	-9.60 ±0.10	-8.09 ±0.06**** P< 0.0001	4
	Emax	100.0 ±0.0	102.5 ±5.1^{NS} P=0.6363	4	100.0 ±0.0	94.0 ±5.4^{NS} P=0.3086	4	100.0 ±0.0	100.9 ±5.8^{NS} P=0.8872	4	100.0 ±0.0	98.6 ±4.0^{NS} P=0.7389	4
Bias Value (G_i VS. β-arrestin2)		0.00 ±0.00	2.25 ±0.15	4	0.00 ±0.00	1.05 ±0.16** P=0.0072	4	0.00 ±0.00	0.48 ±0.32*** P=0.0004	4	0.00 ±0.00	0.91 ±0.23** P=0.0034	4

Supplementary Table 5 | Effects of CCL15 N-terminal truncations on CCR1 (Y291$^{7.43}$A).

NanoBiT results of G protein dissociation and β-arrestin recruitment were normalized to maximal response of CCL15^{M}. N= eight independent experiments, performed with single replicates. Superscripts indicated statistically significant difference (**P<0.01, *P<0.05, and NS was no significance) for every CCL15 truncations vs. CCL15(27-92) as determined by one-way ANOVA. All data were shown as mean±SEM. The data were related to curves shown in **Fig. 4d-e**.

Ligand	G_i Activation		β-arrestin2 Recruitment		Bias Value (G_i VS. β-arrestin2)	n
	log EC50	Emax	log EC50	Emax		
**CCL15(26-92)**	$-8.63\pm0.14^{**}$ P=0.0032	103.0 ± 2.0^{NS} P=0.9790	-9.10 ± 0.10^{NS} P=0..3894	97.8 ± 2.7^{NS} P=0.9921	$-0.93\pm0.15^{**}$ P=0.0067	8
**CCL15(27-92)**	-9.27±0.12	100.0±0.0	-8.79±0.12	100.0±0.0	0.00±0.00	8
**CCL15(28-92)**	-9.25 ± 0.12^{NS} P=0.9999	98.3 ± 5.2^{NS} P=0.9982	-8.89 ± 0.19^{NS} P=0.9833	88.2 ± 3.0^{NS} P=0.1176	-0.07 ± 0.23^{NS} P=0.9986	8
**CCL15(29-92)**	-9.35 ± 0.17^{NS} P=0.9854	98.0 ± 4.1^{NS} P=0.9967	-9.31 ± 0.27^{NS} P=0.1311	$83.0\pm5.9^{*}$ P=0.0115	-0.28 ± 0.28^{NS} P=0.7725	8
**CCL15(30-92)**	-9.03 ± 0.05^{NS} P=0.5359	106.3 ± 5.7^{NS} P=0.7136	-8.40 ± 0.08^{NS} P=0.2095	$85.5\pm4.7^{*}$ P=0..0368	0.25 ± 0.20^{NS} P=0.8323	8
**CCL15(31-92)**	$-8.79\pm0.11^{*}$ P=0.0383	103.1 ± 4.6^{NS} P=0.9745	-8.95 ± 0.07^{NS} P=0.8762	86.9 ± 3.2^{NS} P=0.0703	-0.57 ± 0.20^{NS} P=0.1613	8

Supplementary Table 6 l Effects of CCL15^{M} on wild type CCR1 and mutations on G_i activation and β-arrestin recruitment.

NanoBiT results of G protein dissociation and β-arrestin recruitment for CCR1(wild-type), CCR1(T86$^{2.56}$A/W90$^{2.60}$A) and CCR1(Y113$^{3.32}$F/ Y255$^{6.51}$F). Data shown were normalized to maximal CCR1 (wild-type)/ CCL15^{M} response. N= four independent experiments performed with quadruple replicates. The statistical difference between CCR1 (wild-type) or CCR1(T86$^{2.56}$A/W90$^{2.60}$A) and CCR1(Y113$^{3.32}$F/ Y255$^{6.51}$F) was calculated using two-tailed Student's t-test. Superscripts indicated statistically significant difference (****P<0.0001, ***P<0.001, **P<0.01, *P<0.05, and NS was no significance). All data were shown as mean± SEM. The data were related to curves shown in **Fig. 4f-h**.

		Wild-type	T86$^{2.56}$A/W90$^{2.60}$A	Wild-type	Y113$^{3.32}$F/Y255$^{6.51}$F	n
G_i Activation	log EC50	-9.67±0.36	-9.80±0.26^{NS}	-8.97±0.26	-9.23±0.36^{NS}	4
	Emax	100.0±0.0	81.1±4.0**	100.0±0.0	105.4±3.2^{NS}	4
β-arrestin2 Recruitment	log EC50	-8.78±0.16	-9.32±0.24^{NS}	-8.84±0.19	-7.99±0.16*	4
	Emax	100.0±0.0	78.3±12.6****	100.0±0.0	78.1±1.3****	4
Bias Value (G_i VS. β-arrestin2)		0.00±0.00	-0.38±0.08**	0.00±0.00	1.24±0.16***	4

Supplementary Table 7| Structure-based sequence alignment of residues of the central polar network of class A GPCR with $Y^{7.43}$.

Receptors		Ballesteros–Weinstein GPCR numbering				
Uniport	IUPHAR	2.56	2.60	3.32	6.51	7.43
ccr1_human	CCR1	T	W	Y	Y	Y
ccr3_human	CCR3	T	W	Y	Y	Y
cxcr3_human	CXCR3	T	W	F	Y	Y
apj_human	apelin	T	W	I	Y	Y
cxcr6_human	CXCR6	T	W	Y	F	Y
taar8_human	TAAR8	T	F	D	Y	Y
agtr1_human	AT1R	T	W	V	H	Y
gpr78_human	GPR78	D	T	D	Y	Y
gpr26_human	GPR26	N	T	D	Y	Y
acm1_human	M1R	S	Y	D	Y	Y
acm2_human	M2R	S	Y	D	Y	Y
acm4_human	M4R	S	Y	D	Y	Y
acm5_human	M5R	S	Y	D	Y	Y
acm3_human	M3R	S	F	D	Y	Y
ntr1_human	NTS1R	A	E	R	Y	Y
ntr2_human	NTS2R	G	E	H	Y	Y
trfr_human	TRHR	A	N	Q	Y	Y
hrh1_human	H1R	V	N	D	Y	Y
npbw1_human	GPR7	V	N	D	Y	Y
hrh2_human	H2R	V	S	D	Y	Y
taar6_human	TAAR6	V	S	D	Y	Y
taar9_human	TAAR9	V	S	D	Y	Y
gpr45_human	GPR45	C	T	Y	H	Y
oprd_human	δ-OR	T	Q	D	I	Y
oprk_human	κ-OR	T	Q	D	I	Y
oprm_human	μ-OR	T	Q	D	I	Y
oprx_human	NOP	T	Q	D	V	Y
oxer1_human	OXER1	N	R	L	S	Y
taar2_human	TAAR2	I	S	D	C	Y
taar3_human	TAAR3	I	S	D	C	Y
ada2b_human	α2B-AR	I	S	D	F	Y
5ht1b_human	5HT1B	V	S	D	F	Y
5ht1d_human	5HT1D	V	S	D	F	Y
5ht1e_human	5HT1E	V	S	D	F	Y
5ht1f_human	5HT1F	V	S	D	F	Y
5ht2a_human	5HT2A	V	S	D	F	Y
5ht2c_human	5HT2C	V	S	D	F	Y
5ht5a_human	5HT5A	V	S	D	F	Y
ada1a_human	α1A-AR	V	S	D	F	Y
ada1b_human	α1B-AR	V	S	D	F	Y
ada1d_human	α1D-AR	V	S	D	F	Y
ada2a_human	α2A-AR	V	S	D	F	Y
ada2c_human	α2C-AR	V	S	D	F	Y
taar1_human	TA1R	V	S	D	F	Y
taar5_human	TA5R	V	S	D	F	Y
npbw2_human	GPR8	V	N	D	F	Y
ghsr_human	ghrelin	C	D	S	F	Y
ox1r_human	OX1R	C	S	Q	I	Y
ox2r_human	OX2R	C	T	Q	I	Y
kissr_human	KISSIR	C	T	Q	I	Y
gpr39_human	GPR39	G	E	F	N	Y

galr1_human	GAL1R	C	Q	F	H	Y
galr2_human	GAL2R	C	Q	I	H	Y
galr3_human	GAL3R	C	Q	I	H	Y
hcar1_human	HCAR1	C	R	L	S	Y
ur2r_human	NMUR2	S	I	D	F	Y
ssr1_human	SSTR1	S	L	D	F	Y
ssr4_human	SSTR4	S	V	D	F	Y
mtr1l_human	GPR50	Y	L	T	I	Y
mchr1_human	MCH1R	G	M	D	Y	Y
mchr2_human	MCH2R	G	L	D	Y	Y
gpr61_human	GPR61	L	A	S	Y	Y
gpr62_human	GPR62	I	G	S	Y	Y
c5ar1_human	C5AR1	A	L	I	Y	Y
bkrb2_human	B1R	G	W	I	F	Y
mtlr_human	motilin	G	D	G	F	Y
nmur1_human	NMUR1	G	E	F	F	Y
nmur2_human	NMUR2	G	E	F	F	Y
5ht1a_human	5HT1A	V	A	D	F	Y
5ht2b_human	5HT2B	V	A	D	F	Y
5ht6r_human	5HT6R	V	A	D	F	Y
adrb3_human	β3AR	V	A	D	F	Y
drd4_human	D4R	V	F	D	F	Y
5ht4r_human	5HT4R	V	G	D	F	Y
adrb1_human	β1AR	V	G	D	F	Y
adrb2_human	β2AR	V	G	D	F	Y
5ht7r_human	5HT7R	V	V	D	F	Y
drd2_human	D2R	V	V	D	F	Y
drd3_human	D3R	V	V	D	F	Y
ssr2_human	SSTR2	G	L	D	F	Y
ssr3_human	SSTR3	G	L	D	F	Y
ssr5_human	SSTR5	G	L	D	F	Y
gpr63_human	GPR63	N	A	F	F	Y
cckar_human	CCK1R	C	N	M	I	Y
gpr22_human	GPR22	C	T	V	I	Y
gasr_human	CCK2R	C	T	M	V	Y
hcar2_human	HCAR2	C	L	L	S	Y
hcar3_human	HCAR3	C	V	F	S	Y
mtr1a_human	MT1R	P	V	M	L	Y
mtr1b_human	MT2R	P	I	M	L	Y
gpr31_human	GPR31	C	L	L	C	Y

Residues exactly like CCR1 at the same location are colored as yellow. Positive charged residues are colored as blue. Negative charged residues are colored as red. Uncharged polar residues are colored as green.
