# Supplementary material for: Identification and mechanism of G protein-biased ligands for chemokine receptor CCR1
Source: Nat Chem Biol. 2021 Dec 23;18(3):264–71. doi: 10.1038/s41589-021-00918-z (PMC8885419; doi:10.1038/s41589-021-00918-z)
Supplement: Supplementary file 2 — Reporting Summary [file 41589_2021_918_MOESM2_ESM.pdf]

nature research

Corresponding author(s): Huahao Shen
Yan Zhang
Songmin Ying

Last updated by author(s): Oct 5, 2021

Reporting Summary

Nature Research wishes to improve the reproducibility of the work that we publish. This form provides structure for consistency and transparency in reporting. For further information on Nature Research policies, see our Editorial Policies and the Editorial Policy Checklist.

Statistics

For all statistical analyses, confirm that the following items are present in the figure legend, table legend, main text, or Methods section.

n/a	Confirmed	
☐	☒	The exact sample size (*n*) for each experimental group/condition, given as a discrete number and unit of measurement
☐	☒	A statement on whether measurements were taken from distinct samples or whether the same sample was measured repeatedly
☐	☒	The statistical test(s) used AND whether they are one- or two-sided<br>*Only common tests should be described solely by name; describe more complex techniques in the Methods section.*
☒	☐	A description of all covariates tested
☒	☐	A description of any assumptions or corrections, such as tests of normality and adjustment for multiple comparisons
☐	☒	A full description of the statistical parameters including central tendency (e.g. means) or other basic estimates (e.g. regression coefficient) AND variation (e.g. standard deviation) or associated estimates of uncertainty (e.g. confidence intervals)
☐	☒	For null hypothesis testing, the test statistic (e.g. *F*, *t*, *r*) with confidence intervals, effect sizes, degrees of freedom and *P* value noted<br>*Give P values as exact values whenever suitable.*
☒	☐	For Bayesian analysis, information on the choice of priors and Markov chain Monte Carlo settings
☒	☐	For hierarchical and complex designs, identification of the appropriate level for tests and full reporting of outcomes
☒	☐	Estimates of effect sizes (e.g. Cohen's *d*, Pearson's *r*), indicating how they were calculated

Our web collection on statistics for biologists contains articles on many of the points above.

Software and code

Policy information about availability of computer code

Data collection	Automated data collection on the Titan Krios was performed using the serialEM software; Data collection of flow cytometry was performed using the CytoFlex Cytometer; Data collection of NanoBiT, Glosensor and BRET assays was performed using the Spark Multimode microplate reader (Tecan).
Data analysis	The following software was used in this study: PyMol 2.5, Bsoft 2.0.7, MotionCor2 1.3.2, Gctf 1.18, RELION 3.0.8, UCSF Chimera 1.15, UCSF ChimeraX 1.2.5, Coot 0.9.4, Phenix 1.18, NAMD 2.13, VMD 1.9.3, CytoExpert 2.0,Grapad Prism 7, Origin 2018.

For manuscripts utilizing custom algorithms or software that are central to the research but not yet described in published literature, software must be made available to editors and reviewers. We strongly encourage code deposition in a community repository (e.g. GitHub). See the Nature Research guidelines for submitting code & software for further information.

Data

Policy information about availability of data

All manuscripts must include a data availability statement. This statement should provide the following information, where applicable:
- Accession codes, unique identifiers, or web links for publicly available datasets
- A list of figures that have associated raw data
- A description of any restrictions on data availability

Cryo-EM maps of apo CCR1-Gi, CCL15M-CCR1-Gi and CCL15L-CCR1-Gi complexes have been deposited in the Electron Microscopy Data Bank under accession codes EMD-32020, EMD-32021 and EMD-32022, respectively. The atomic coordinates of apo CCR1-Gi, CCL15M-CCR1-Gi and CCL15L-CCR1-Gi complexes have been deposited in the Protein Data Bank under accession codes 7VL8, 7VL9 and 7VLA, respectively. All relevant data are available from the authors and/or included in the manuscript or Supplementary Information.

Field-specific reporting

Please select the one below that is the best fit for your research. If you are not sure, read the appropriate sections before making your selection.

☒ Life sciences ☐ Behavioural & social sciences ☐ Ecological, evolutionary & environmental sciences

For a reference copy of the document with all sections, see nature.com/documents/nr-reporting-summary-flat.pdf

Life sciences study design

All studies must disclose on these points even when the disclosure is negative.

Sample size	For structural determination, images were collected until the resolution and 3D reconstruction converges. For functional assays, no statistical approaches were used to predetermine the sample size. We performed at least three independent experiments as indicated in related figure legends and methods, which were projected to provide adequate power to detect statistically significant differences.
Data exclusions	No data was systematically excluded. The procedure of generating 3D maps from cryo-EM particles invloved sorting of particles that were damaged or false-picked and unlikely to be refined correctly. This was implemented in RELION-3.0-beta2. In analysis of functional results, outlines fell outside three standard deviations from the mean were excluded from further analyses.
Replication	All functional assays were performed in at least three independent experimental replicates and reliably reproduced within one month. All MD-sitmulation analysis were performed in five replicates. All attempts at replication of signaling assays succeeded. There was no attempt to replicate cryo-EM data. This data involves averaging of tens of thousands of particles.
Randomization	Randomization was not relevant to this study, as protein samples are not required to be allocated into experimental groups in protein structural studies, and no animals or human research participants were involved in this study.
Blinding	Binding was not relevant to this study. For structural determination, protein samples were not required to be allocated into experimental groups. For functional analysis, blinding is not necessary since the data was collected automatically by the microplate reader.

Reporting for specific materials, systems and methods

We require information from authors about some types of materials, experimental systems and methods used in many studies. Here, indicate whether each material, system or method listed is relevant to your study. If you are not sure if a list item applies to your research, read the appropriate section before selecting a response.

Materials & experimental systems

n/a	Involved in the study	
☐	☒	Antibodies
☐	☒	Eukaryotic cell lines
☒	☐	Palaeontology and archaeology
☒	☐	Animals and other organisms
☒	☐	Human research participants
☒	☐	Clinical data
☒	☐	Dual use research of concern

Methods

n/a	Involved in the study	
☒	☐	ChIP-seq
☐	☒	Flow cytometry
☒	☐	MRI-based neuroimaging

Antibodies

Antibodies used	APC anti-human CD191(CCR1) Antibody (Biolegend, Catalog #362907), used in 1:1000 dilution;<br>PE anti-DYKDDDDK Tag antibody (Biolegend, Catalog #637310), used in 1:1000 dilution.
Validation	All antibodies were commercially obtained and validation reports were available on the supplier website:<br>APC anti-human CD191(CCR1) Antibody: https://www.biolegend.com/en-us/products/apc-anti-human-cd191-ccr1-antibody-9957?GroupID=BLG13118<br>PE anti-DYKDDDDK Tag antibody: https://www.biolegend.com/en-us/products/pe-anti-dykddddk-tag-antibody-9383?GroupID=GROUP26

Eukaryotic cell lines

Policy information about cell lines

Cell line source(s)	HEK 293T cells were purchased from Cell Bank at the Chinese Academy of Sciences.<br>SF9 cells were purchased from Expression Systems (Cat 94-001S).<br>High Five cells were purchased from Expression Systems (Cat 94011S).

	Human monocytic THP-1 cells were purchased from ATCC (Cat ATCC-TIB-202).
Authentication	All of the cells lines mentioned above are maintained by the supplier. No additional authentication was performed by the authors of this study.
Mycoplasma contamination	The above cell lines were negative for Mycoplasma contamination.
Commonly misidentified lines (See ICLAC register)	No commonly misidentified cell lines were used.

Flow Cytometry

Plots

Confirm that:

☒ The axis labels state the marker and fluorochrome used (e.g. CD4-FITC).

☒ The axis scales are clearly visible. Include numbers along axes only for bottom left plot of group (a 'group' is an analysis of identical markers).

☒ All plots are contour plots with outliers or pseudocolor plots.

☒ A numerical value for number of cells or percentage (with statistics) is provided.

Methodology

Sample preparation	THP-1 cells were fluorescently labeled by using the APC anti-human CD191 (CCR1) antibody. Transfected HEK 293T cells, which overexpressed CCR1, were fluorescently labeled by using the PE anti-DYKDDDDK antibody.
Instrument	CytoFLEX LX Flow Cytometer equipped with 488/633/405/375/561 lasers, Beckman Coulter.
Software	Flow cytometry data were collected by CytoFlex Cytometer and analyzed by CytoExpert2.0.
Cell population abundance	Moderate
Gating strategy	For THP-1 cells, gating using PB450 versus FSC-A was performed to exclude dead cells; FSC-A versus SSC-A was performed to exclude cell debris. And FSC-H versus FSC-A was used to distinguish single cells. For HEK 293T cells, the gate strategy was similar with that used in THP-1 cells as described above, except that the successful transfected HEK 293T cells were further identified with high FITC fluorescence.

☒ Tick this box to confirm that a figure exemplifying the gating strategy is provided in the Supplementary Information.
